# Humoral Response in Cattle Vaccinated with the Heterologous Sheeppox Virus Vaccine for Protection Against Lumpy Skin Disease: A Field Study

**DOI:** 10.3390/vaccines13121221

**Published:** 2025-12-03

**Authors:** Anna M. Lyapina, Natalya V. Kichemazova, Maxim S. Lavrukhin, Yuri V. Saltykov, Sergey S. Zaytsev, Olga S. Larionova, Valentina A. Feodorova

**Affiliations:** 1 Fundamental and Applied Research Lab, Saratov State University of Genetics, Biotechnology and Engineering Named After N.I. Vavilov, 335 Sokolovaya Str., 410005 Saratov, Russia; 2 Department for Microbiology and Biotechnology, Institute for Biotechnology, Saratov State University of Genetics, Biotechnology and Engineering Named After N.I. Vavilov, 335 Sokolovaya Str., 410005 Saratov, Russia; 3 Institute for Veterinary Medicine and Pharmacy, Saratov State University of Genetics, Biotechnology and Engineering Named After N.I. Vavilov, 335 Sokolovaya Str., 410005 Saratov, Russia

**Keywords:** capripoxvirus, heterologous vaccine, LSDV, SPPV, humoral immune response, ELISA, immunoblotting, P32, immunoreactive proteins, cattle

## Abstract

Background/Objectives: Lumpy skin disease (LSD) is a notifiable disease due to a marked potency for rapid spread and a significant negative economic impact on agriculture worldwide. As such, vaccination is considered the most effective way to control the disease in endemic countries, and the serological response to homologous LSDV-based vaccines is widely investigated. However, less is known about the seroconversion and duration of the immune response that is elicited by live attenuated heterologous vaccines based on the Sheeppox virus (SPPV) used for LSD prevention. This study aimed to investigate the humoral immune response in cattle immunized with a heterologous SPPV-based vaccine in the field. Methods: Commercial ELISA, based on P32 protein, as well as immunoblotting, were used to assess the antibody response in 6-month-old, 17-month-old, and older animals before and after the first immunization and revaccination. Results: Only a secondary immune response was detected when using commercial ELISA in revaccinated animals in each of the groups (83.3% and 30%, respectively). A comparative bioinformatic analysis proved a marked polymorphism of P32, derived from LSDV/SPPV/GTPV, which potentially resulted in negative responses in the ELISA. However, immunoblotting revealed a 100% seroconversion in vaccinated animals after the first vaccination and revaccination. Notably, specific antibodies were found in the sera of 80% of 6-month-old calves before the first vaccination, which had probably been passively transferred from their mothers, who were multiply vaccinated with SPPV-based vaccines. Conclusions: Several immunodominant antigens were able to induce a humoral immune response in cattle to the SPPV-based vaccine after passive and active immunization, serving as promising markers for a humoral immune response to heterologous vaccines.

## 1. Introduction

Lumpy skin disease (LSD) is a vector-borne disease of cattle that is caused by a DNA-containing Lumpy skin disease virus (LSDV), also known as Neethling virus, which belongs to the genus *Capripoxvirus* (CapPV) of the family *Poxviridae*. The disease is one of the most important viral diseases of cattle, causing huge economic losses in the livestock industry [1]. LSDV can be transmitted through direct or indirect contact between animals, including consumption of contaminated food or water, and mechanically by blood-sucking insects [2,3,4]. Clinically, LSD is mainly characterized by fever, skin nodules, lymphadenitis, and lesions of the ocular, oral, and nasal mucous membranes [5,6]. The disease causes significant losses for the agricultural sector of the economy: milk yields are reduced, skin is damaged, abortions often occur in pregnant animals, and the disease can cause temporary or permanent sterility. Generally, LSD tends to have a mortality of <10% and a morbidity of 0–90% [7]. Due to its marked potency for rapid spread and the fact that it is a significant barrier for trade in live cattle and livestock products, LSD, along with two other CapPV-related viral infections, Sheeppox (SPP) and Goatpox (GTP), has been specified by the Office International des Epizooties (OIE) (since 2003, the World Organization for Animal Health (WOAH)) as a notifiable transboundary disease [1].

Firstly, LSD was described in 1929 in Northern Rhodesia (Zambia). Then, the disease spread to most countries of Sub-Saharan Africa and later towards the Mediterranean basin and the Middle East. Since 2013, LSD has reached the Middle East and southeast Europe, affecting Turkey (2013–2019), Azerbaijan (2014), Armenia (2015), Greece (2015–2017), Russia (2015–2019), Albania (2016–2017), Bulgaria (2016), Montenegro (2016), Serbia (2016), Macedonia (2016–2017), and Georgia (2016, 2018) [8]. In 2019, LSD entered East Asia and, to date, is prevalent in many Asian countries, including China, India, Bangladesh, Thailand, and Vietnam [8,9,10]. The first case of LSD in Russia was registered in the territory of the Republic of Dagestan in 2015. Then, 17 LSD outbreaks were detected in three regions of the Russian Federation during the summer and autumn of that year. Further, in 2016, 313 LSD outbreaks were officially registered in 16 regions, and 204 LSD outbreaks in 23 different regions of the country were reported between 2017 and 2024 [11,12,13]. In the Saratov region, which is located in southeastern Europe and borders the Republic of Kazakhstan, at least 30 outbreaks of LSD were officially reported between 2017 and 2019 [14,15,16,17].

Nowadays, vaccination of farm animals is considered the most effective, sustainable, and cheapest way to control the spread of LSD in endemic countries. Currently, only live attenuated (either homologous or heterologous) vaccines against LSD based on Capripoxvirus are used for immunizing cattle worldwide [18,19]. Homologous vaccines are commonly the LSDV strain of the Neethling type, which was initially isolated in South Africa in the 1950s. Despite their doubtless efficacy in LSD prophylaxis, these vaccines often cause serious post-vaccination complications or adverse reactions, resembling disease symptoms, which have resulted in a number of diagnostic difficulties [18,20,21,22,23,24,25]. Homologous LSDV vaccines provoked a serological immune response in vaccinated animals, which was very similar to that in LSDV-infected cattle. Thus, serological investigation, while being crucial for proper disease surveillance, control, and eradication efforts, could seriously complicate the differentiation between infected and vaccinated animals (DIVA) [22,23,26]. Moreover, a live attenuated vaccine can replicate in vaccinated animals, which increases the risk of transmitting the virus to healthy animals [18,21]. Heterologous vaccines are based on attenuated strains of two other CapPV representatives, Sheeppox virus (SPPV) and Goatpox virus (GTPV) [11,19,27]. All three viruses, LSDV, SPPV, and GTPV, have antigenic homology and could provide cross-protection from one another. Thereby, all of them are considered to be promising vaccines against LSD [28,29]. Furthermore, heterologous vaccines that are based mainly on SPPV, and less frequently on GTPV, are now widely used for LSD prophylaxis as they provide a high level of population immunity, measured as the seroconversion rate of up to 80% or more in vaccinated cattle [26,30,31,32,33,34,35]. According to a number of reports, seroconversion in cattle vaccinated against LSD with both homologous and heterologous vaccines could be available via ELISA after 3–4 weeks post-immunization, while the spectrum of specific serum antibodies may vary [33,34,35,36,37,38,39]. In the Russian Federation, only live attenuated heterologous vaccines based on SPPV strains are used for LSD prevention in cattle. According to the official statistics, active regular annual immunization of cattle with an SPPV-based vaccine during the last several years has led to a significant decrease in the number of LSD outbreaks, up to a single case in the last year, 2024, in the Republic of Khakassia. Unfortunately, the information on seroconversion rates, duration, and specificity of immune response in cattle following immunization with an SPPV-based vaccine in the Russian Federation is still lacking. However, it is critical to provide a good serological tool that is suitable for monitoring the immune response in cattle to a heterologous vaccine against LSD. The aim of this study was to investigate the humoral immune response caused in cattle by a heterologous SPPV-based vaccine, which is actively used for the annual immunization of cattle against LSD.

## 2. Materials and Methods

### 2.1. Study Area

Sampling for the investigation of seroconversion of cows that were annually vaccinated with a heterologous SPPV-based vaccine against LSD was conducted in cattle from beef households. The settings were located in the territory of the Saratov region, in which at least 30 outbreaks of LSD were officially reported during 2017–2019 [14,15,16,17]. The specimens were collected in cattle from the Engelssky District, which is located about 287.0 km southeast of the border between the Russian Federation and the Republic of Kazakhstan (Figure S1A).

### 2.2. Animals

A small convenience sampling with random selection was carried out for this study. Two groups of animals, females (of the Holstein breed), who were vaccinated with the live, dry, cultural vaccine against Sheeppox and LSD, known as the ‘SheepPox-LSD vac’ (SPPV-based vaccine), with strain/serotype ARRIAH, the clone of the well-known NISKHI strain [40] (Federal Center for Animal Health, Vladimir, Russia, https://shop.arriah.ru/catalog/vaktsiny/ospa-ovets-i-zaraznogo-uzelkovogo-dermatita-krs-kulturalnaya-sukhaya-shippoks-lsd-vak-fl-500doz/ (accessed on 4 October 2025)), were enrolled: 6-month-old naive suckling calves that were vaccinated for the first time and revaccinated one year later (group A, n = 30) under the Federal annual vaccination campaign, and a group of cattle aged 17 months and older (group B, n = 30), which was primarily vaccinated according to the same immunization schedule during the emergency vaccination campaign. Before vaccination, revaccination, and on the day when the SPPV-based vaccine was introduced, all animals of the experimental groups were healthy and showed no clinical signs of either LSD or any other disease. Also, during the observation period, all the animals were kept under the same or similar conditions according to the National Requirements (https://fsvps.gov.ru/files/prikaz-minselhoza-rossii-ot-21-10-2020-622-veterinarnye-pravila-soderzhanija-krupnogo-rogatogo-skota-v-celjah-ih-vosproizvodstva-vyrashhivanija-i-realizacii/, accessed on 4 October 2024).

### 2.3. Sample Collection

The study protocol was approved by the Local Ethics Committee of the Saratov State University of Genetics, Biotechnology and Engineering, named after N.I. Vavilov (approval # 20241011-03 from 11 October 2024), and followed the national legislation and the rules stated in the ARRIVE guidelines 2.0. Permission and informed consent from the beef households’ owners were obtained. All methods were performed in accordance with the relevant guidelines and regulations of the Russian Federation.

Blood sampling was conducted by veterinarian professionals. Blood specimens from each of the animals were collected 1 day before and on day 28 post-vaccination and post-revaccination in field conditions. Each sample of 10 mL was taken from the coccygeal vein into a sterile BD Vacutainer clot activator tube without anticoagulant by vein puncture and delivered to the lab. Sera were separated by centrifugation at 3000 rpm (1660 g) and then stored in cryovials at −20 °C until used for serological studies.

The following sera samples were available for the analysis: before and one month after the first vaccination, before and one month after revaccination, and three months post-revaccination (Figure S1B). A total of 300 samples, taken from both groups of animals at different time points before and after vaccination/revaccination, were examined within the study period. For the controls, the blood samples from either a vaccinated cow with LSD [17] (positive control) or a clinically healthy, naive, unvaccinated cow (negative control) were collected according to the same protocol. For DNA extraction following PCR testing for the presence of LSDV genetic material, blood specimens were taken into test tubes with EDTA-K3.

### 2.4. DNA Extraction and PCR Amplification

The DNA samples were extracted from the individual whole-blood specimens of the investigated cattle at each of the sampling time points using the DNeasy Blood and Tissue Kit (QIAGEN GmbH, Hilden, Germany) according to the manufacturer’s instructions. Detection of the CapPVs genome from the total DNA samples was done using a GPCR gene targeting PCR reaction for identification and intraspecific discrimination between LSDV, SPPV, and GTPV. For this reason, the primers and protocol, initially designed by Le Goff et al. [41] and Ireland and Binepal [42], were used as previously reported [17]. The total DNA derived from the blood samples of a cow with confirmed LSD [17] was utilized as the positive control for the presence of LSDV genetic material in infected animals alone. The total DNA from clinically healthy, naive, unvaccinated cows from another region was used as a negative control.

### 2.5. ELISA

Detection of CapPV-specific antibodies in individual bovine antisera was done using the commercially available ELISA (ID vet®Capripox Double Antigen Multi-species, Montpellier, France), IDvet-ELISA, according to the manufacturer’s instructions. The interpretation of the ELISA results was based on the calculation of the S/P ratio (a S/P ratio of ≥30% of the sample was considered positive for the presence of CapPV-specific antibodies). Bovine sera from three intact, unvaccinated animals from another region with no history of vaccination against LSD were used as negative controls. The serum from a vaccinated cow with confirmed LSD [17] was also used as a positive control.

### 2.6. Immunoblotting

To identify the spectrum of SPPV proteins that reacted with the bovine IDvet-positive and IDvet-negative sera before and after vaccination and revaccination, the immunoblotting technique was used. A routine protocol was implemented as previously described [43]. The whole-cell lysates of the SPPV vaccine strain NISKHI (clone B-5/96) were obtained after purification and inactivation with 0.05% β-propionlactone according to the protocols [44,45] and resolved in 12.5% SDS-PAGE. Separated SPPV proteins were transferred to a nitrocellulose membrane (NCM) with a pore size of 0.22 μm (Bio-Rad Laboratories, Hercules, CA, USA) by electroblotting (semi-dry blot transfer system FTB95, Guangzhou Daoyi Science and Technology Co., Ltd., Guangzhou, China). Each membrane was carefully washed in PBS and blocked for 1.5 h with 1% non-fat dry milk in PBS. Then, the membranes were incubated with bovine antisera diluted at 1:10 in 1% non-fat dry milk in PBS that was supplemented with 0.05% Tween-20 (PBST) at +4 °C for 18–20 h following washing four times. Anti-Bovine IgG (H+L), F(ab′)2 fragment-Peroxidase antibodies produced in rabbit (Sigma-Aldrich, St. Louis, MO, USA) at a dilution of 1:2000 in 1% non-fat dry milk in PBST were used as secondary antibodies and probed with the membranes for 2 h at 25 °C. After five washings with PBST, the membranes were incubated with the relevant TMB substrate (Sigma-Aldrich, USA) for 20 min. The reaction was stopped by washing with deionized water and analyzed visually to detect a positive reaction.

### 2.7. CapPV Strains and P32 Sequence Analysis

All the representative CapPV complete genome sequences of SPPV, LSDV, and GTPV were obtained from the NCBI database (https://www.ncbi.nlm.nih.gov/ (accessed on 26 September 2024)) and are listed in Table S1. The nucleotide and amino acid sequences of the LW074 open reading frame (ORF), expressed initially, before the beginning of the 2000s, in the LSDV Neethling vaccine LW 1959 strain (Acc. No. OM793609.1) as P35 and is currently known as the P32 protein (P32 protein) [46,47,48,49], were obtained from the publicly available NCBI database (Acc. No. AF409138.1), and used as a reference. The whole genome of the SPPV strain NISKHI (Acc. No. AY077834.1) was annotated utilizing the NCBI reference strain SPPV 17077-99 (Acc. No. NC_004002.1) with the GaTU tool [50]. To obtain the nucleotide sequence of the SPPV strain NISKHI P32 protein, the complete SPPV NISKHI genome was aligned along the P32 sequence (ORF LW074) from the LSDV Neethling vaccine strain LW 1959 by MEGA 11 [51], followed by verification through the annotated one in the genome of the SPPV strain NISKHI by MEGA11 [51]. The P32 nucleotide and amino acid sequences of the SPPV vaccine strain NISKHI were aligned versus the relevant sequences of the reference LSDV Neethling vaccine LW1959 and other representative CapPV strains (n = 26, enumerated in Table S2) by MultAlin software, v.5.4 (http://multalin.toulouse.inra.fr/multalin/, accessed on 24 September 2024). Homologs to P32-related nucleotide sequences were identified by BLAST (https://blast.ncbi.nlm.nih.gov/Blast.cgi, accessed on 24 September 2024). A phylogenetic analysis of the P32 gene sequences that were derived from the SPPV vaccine strain NISKHI, the reference LSDV Neethling vaccine LW1959, and other representative CapPV strains was performed using the Neighbor-Joining method in MEGA 11 [51].

### 2.8. In Silico Prediction of Linear and Conformational B- and T-Cell Epitopes for P32 Protein

A panel of B-cell linear and conformational epitopes for the P32 protein derived from either the SPPV vaccine strain NISKHI or the reference LSDV Neethling vaccine LW1959 was predicted by software ElliPro NetMHC v.3.0 (http://tools.iedb.org/ellipro/, 5 August 2025) [52] on the basis of the relevant protein sequences and three-dimensional (3D) models, as reported [53,54]. T-cell epitope composition (MHC I class) [55] was predicted for P32 amino acid sequences from the SPPV strain NISKHI and the LSDV Neethling vaccine strain LW 1959 by the IEDB server tool NetMHCpan v.4.1 (https://www.iedb.org, 5 August 2025). Individual B- and T-cell epitope characteristics, such as immunogenicity, antigenicity, allergenicity, and toxicity, were uncovered using ElliPro NetMHC v.3.0 (http://tools.iedb.org/ellipro/, 5 August 2025), the VaxiJen v.2.0 server (http://www.ddg-pharmfac.net/vaxijen/VaxiJen/VaxiJen.html, 8 August 2025), AllerCatPro v.2.0 (https://allercatpro.bii.a-star.edu.sg/, 8 August 2025) and ToxinPred v.1.0 (http://crdd.osdd.net/raghava/toxinpred, 5 August 2025) web servers [52,56,57,58,59].

### 2.9. Statistical Analysis

GraphPad Prism version 7.0 (GraphPad software, San Diego, CA, USA) was used for statistical analysis and data visualization. Fisher’s exact test was implemented to compare seropositivity to CapPV antigen between groups A and B. Spearman’s rank correlation coefficient was used for the assessment of correlations. A *p*-value < 0.05 was defined as significant.

## 3. Results

### 3.1. PCR

No CapPVs (either LSDV or SPPV, or GTPV) genetic material was found in the specimens from cattle that were initially unvaccinated and further vaccinated with the SPPV-based vaccine (Table 1).

### 3.2. Detection of CapPV Seropositive Animals by IDvet-ELISA

**Figure 1:**
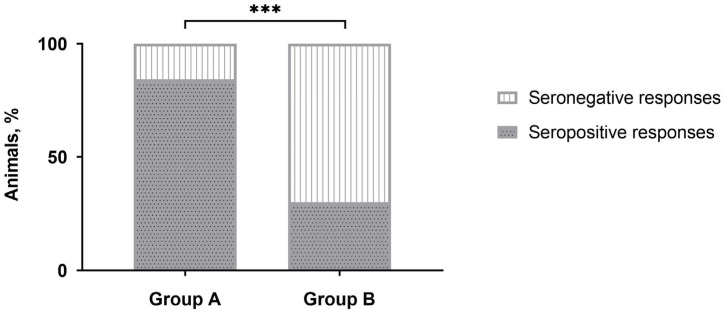
Number of seropositive responses detected by IDvet-ELISA in either group A or group B of cattle on day 28 post-revaccination with the SPPV-based vaccine. *** *p* < 0.001, Fisher’s exact test.

No positive responses were detected by IDvet-ELISA during the majority of sampling points (Table 1). Only after revaccination, the majority of vaccinated animals (more than 80%) were seropositive in group A, while only a third of the animals in group B demonstrated positive responses. However, after three to 12 months, the number of seropositive animals was nullified. No positive reactions were registered with the sera of unvaccinated naive animals in control group C. Interestingly, the number of CapPV-antibody positive cattle detected in group B by IDvet-ELISA on day 28 post-revaccination was almost 2.8 times lower than in group A (Table 1), and this difference was significant (Figure 1).

**Figure 2:**
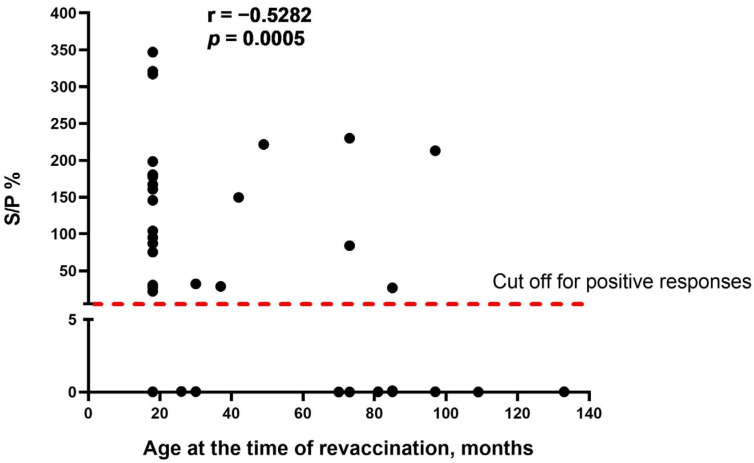
Correlation between the age of cattle at the time of revaccination and antibody titers (S/P %) detected in IDvet-ELISA. S/P % > 30% are considered positive responses. The assessment of relationships between the age of revaccination and antibody response presented as S/P % was performed using the Spearman correlation analysis.

As the obvious variable factor that differed between the two study groups was the age of cattle at the time of revaccination, we assumed that age is one of the main factors influencing the seroconversion rate in cattle vaccinated with the SPPV-based vaccine. We found that there was a moderate negative correlation between the two variables (r = −0.5282, *p* = 0.0005), indicating that the age of revaccinated cattle was inversely associated with the number of seropositive responses in ELISA (Figure 2).

### 3.3. Detection of CapPV Seropositive Animals by Immunoblotting

According to the results of immunoblotting, the majority of calves in group A were seropositive already at the first sampling point. The number of positive animals was slightly higher one year post-vaccination. Vaccination and revaccination with the SPPV-based vaccine resulted in the absolute seroconversion of all immunized animals (Table 1). In contrast, all the animals in group B were seronegative before and after the first vaccination. However, after revaccination, all the immunized animals were seropositive throughout the observation period. No positive reactions in this assay were registered in group C with the sera of unvaccinated naive animals.

### 3.4. Spectrum of Immunoreactive Antigens Identified by Immunoblotting

#### 3.4.1. IDvet-ELISA Negative Sera

**Figure 3:**
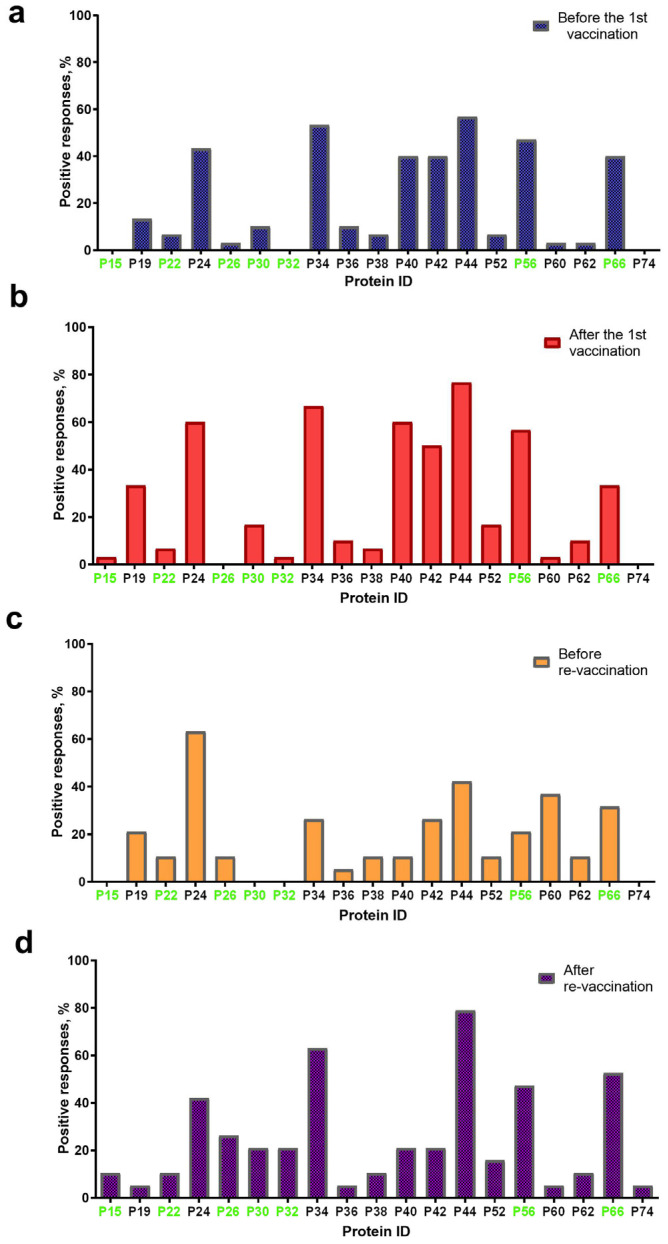
Spectrum of immunoreactive proteins identified by immunoblotting of the whole-cell lysates of the SPPV strain NISKHI with the bovine antisera of group A: (**a**) before vaccination; (**b**) after the first vaccination; (**c**) before revaccination; and (**d**) after revaccination with the SPPV-based vaccine. Immunoreactive proteins identified in the serum of a vaccinated animal with LSD are colored in green.

Overall, 19 proteins with a molecular weight (mol. wt) in the range of 15 ± 2 kDa–74 ± 2 kDa were visualized on blottograms with the antisera of animals in group A either before or post the first immunization with the SPPV-based vaccine (Figure 3). At least 16/19 proteins were detected in blottograms with the antisera of animals before the first vaccination (Figure 3a). Among them, the 24-, 34-, 40-, 42-, 44-, 56-, and 66-kDa proteins (P24, P34, P40, P42, P44, P56, and P66, respectively) gave positive reactions with the antisera of 40–57% animals. The 19-, 30-, 36-kDa proteins (P19, P30, and P36 kDa, respectively) interacted with the serum antibodies of approximately 10–13%, and the 22-, 26-, 38-, 52-, 60-, and 62-kDa proteins (P22, P26, P38, P52, P60, and P62, respectively) of few animals.

After the first vaccination, 16/19 proteins were also detectable with certain changes in their spectrum (Figure 3b). Thus, no positive reaction with P26 was seen on the blottograms, while 15-kDa and 32-kDa proteins (P15 and P32, respectively) were easily revealed by the antiserum of a single cow. In contrast, the number of bovine antisera containing antibodies to several proteins, P19, P24, P30, P34, P40, P42, P44, P52, P56, and P62, significantly increased up to 2.5-fold. Six proteins, P24, P34, P40, P42, P44, and P56, could induce specific Abs in more than 50–77% of the vaccinated cattle, being, probably, immunodominant antigens for the SPPV-based vaccine used. Two proteins, P19 and P66, were identified on the blottograms of about 30% of vaccinated animals, two other proteins, P30 and P52, in about 17% cases, while the rest of the proteins provided a smaller number of positive reactions (no more than 3–10%). One year later, before revaccination, 15/19 proteins were successfully recognized by the bovine immune antisera, although a certain difference in the number of seropositive animals was registered (Figure 3c). Indeed, only P24 could still be detected by the antisera of more than 60% singly vaccinated animals, while Abs to the rest of the immunodominant proteins, P34, P40, P42, P44, and P56, were seen in a markedly smaller number of bovine antisera (from 10.5% to 42.1%). Two proteins, P60 and P66, were detectable with the antisera of more than 30% of seropositive animals. The remainder of the proteins could be immunoreactive with the antisera derived from approximately 5.0–10.5% of the vaccinated cattle. No positive reactions were registered with P15, P30, and P32. No immunoreactive proteins were revealed in the sera of cattle in group B either before or post-vaccination, or in the unvaccinated naive animals of group C that were used as the negative control. In contrast, seven immunoreactive proteins, P15, P22, P26, P30, P32, P56, and P66, were identified in the serum of a vaccinated animal with confirmed LSD in group C (Figure 3).

#### 3.4.2. IDvet-ELISA-Positive Sera

After revaccination, which produced a pronounced serological immune response that was detectable in immunoblotting, in 100% of the vaccinated animals in group A (Table 1), 19/19 proteins were recognized by the bovine antisera (Figure 3d). The same five proteins, P24, P34, P44, P56, and P66, were the most immunoreactive, providing a positive reaction on the majority of the blottograms (42–79%). Several proteins, P26, P30, P32, P40, P42, and P52, could interact with serum antibodies in about 15–25% of the revaccinated animals. The remainder of the proteins were also able to produce positive reactions, although with a markedly smaller number of immune antisera, up to 10%. 

**Figure 4:**
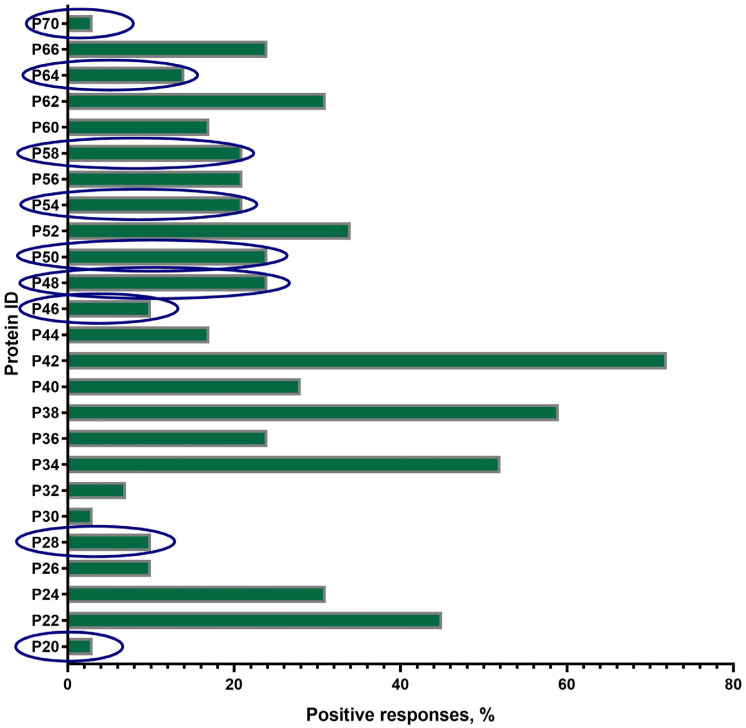
Spectrum of immunoreactive proteins identified by immunoblotting of the whole-cell lysates of the SPPV strain NISKHI with the bovine antisera of group B on day 28 post-revaccination with the SPPV-based vaccine. Novel proteins, antibodies to which were detected specifically in group B, were absent in group A, and are in the blue oval.

A total of 25 proteins with a mol. wt. in the range of 20 ± 2 kDa–70 ± 2 kDa were visualized on the blottograms with the antisera of animals in group B just after revaccination with the SPPV-based vaccine (Figure 4). Overall, all of these 25 proteins (25/25, 100%) were immunoreactive and totally recognized by the antisera from all animals (30/30, 100%) in group B. However, the number of proteins that were able to produce seroconversion certainly differed in individual animals. Thus, at least three proteins, P34, P38, and P42, gave a positive reaction with the antisera from more than 50–70% animals. P22, P24, P52, and P62 were immunoreactive with the antisera of 30–45% animals, while P36, P40, P44, P56, P60, and P66 reacted with only 15–25% of the antisera investigated. No positive reaction was registered with P15, P19, and P74. Surprisingly, nine proteins—antibodies to which were detected specifically in group B—were absent in group A, such as P20, P28, P46, P48, P50, P54, P58, P64, and P70.

#### 3.4.3. The Panel of the Most Immunoreactive Antigens

The majority of proteins (14/19), except P15, P26, P30, P32, and P74, were immunoreactive during all four sampling time points in group A. The number of animals that were seropositive to other proteins could differ during the observation period (Figure 3). Some of these gave constant positive reactions with antisera, from 30–60% up to about 80% of animals (P24, P34, P44, P56, and P66) or no more than 15% (P22, P36, P38, P52, P60, and P62). The number of positive responses to several proteins increased slowly but surely (P26 and P30) or was registered only after the immunization (the first vaccination and the revaccination) (P15, P32, P52, and P56). Some proteins, P19, P40, and P42, were recognized by the antisera of animals exclusively after the first vaccination or only after revaccination (P74). Only three proteins, P34, P38, and P42, were immunoreactive during the observation period in 50–70% of the revaccinated animals in group B. All the other proteins also gave positive reactions, but in a smaller number of animals (1/30–13/30, 3.3–43.3%) (Figure 4). Finally, P24, P34, P40, P42, P44, P56, and P66 were immunoreactive with the majority of antisera from the animals in both groups A and B (Figure 3 and Figure 4). In fact, in group A, P32-specific antibodies were found by immunoblotting in the antisera of only individual cattle in two sampling points, namely just after the first vaccination in a sole animal (1/30, 3.3%) and following revaccination in a few animals (6/30, 20%) (Figure 3b,d). Similarly, the antibodies that were able to recognize P32 in immunoblotting with the bovine antisera in group B were only detected in a few animals (2/30, 6.6%) (Figure 4).

### 3.5. Polymorphism of P32 Protein

**Figure 5:**
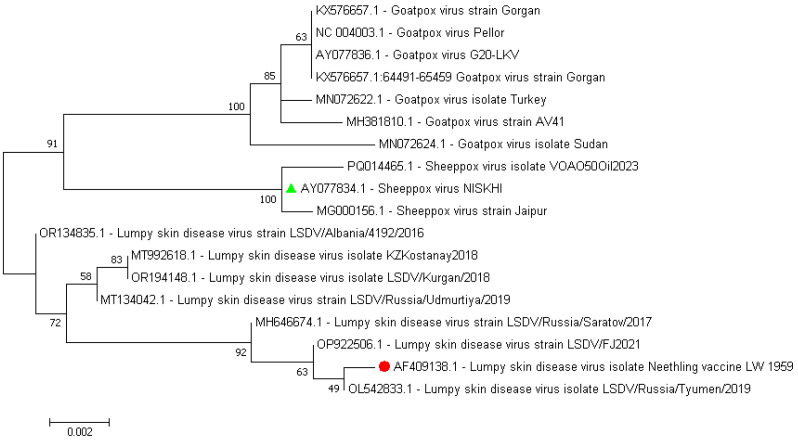
Dendrogram of the nucleotide sequences of P32 derived from the CapPV representatives, such as LSDV, GTPV, and SPPV strains. The colored green triangle and red circle represent the sequences from the SPPV vaccine strain NISKHI and the reference LSDV Neethling_vaccine_LW_1959, respectively.

Comparative alignment of the nucleotide sequences of the P32 protein that was derived from the whole genome sequences of the SPPV vaccine strain, NISKHI, versus the reference LSDV Neethling_vaccine_LW_1959 and the majority of the LSDV representatives revealed 18 substitutions that resulted in 12 synonymous and five nonsynonymous replacements in the relevant P32 amino acid sequence in positions 49 (F→L, F49L), 62 (F→L, F62L), 98 (V→A, V98A), 132 (L→S, L132S), and 134 (T→I, T134I) and the insertion of a single amino acid, Aspartate, in position 55 (D55ins) (Figure S2, Table S2). All identical mutations were regularly identified in each of the P32 amino acid sequences derived from the seven SPPV representative strains, while being absent in other CapPVs, both LSDV and GTPV. An additional single SNP in position 141 (E→K, E141K) was revealed in the SPPV Jaipur/INDIA-1981. No substitution in position 98 (A98A) in the amino acid sequences of the P32 protein of the SPPV strain NISKHI and two LSDV strains, LSDV NI-2490 and LSDV SERBIA/Bujanovac/2016, was detected. Further comparison of the P32 protein amino acid and nucleotide sequences of the SPPV vaccine strain NISKHI with the homologous sequences of the same P32 of the GTPV representative strains (n = 12) also showed a certain polymorphism. Thus, nine substitutions in positions 23 (D26G, D→G), 46 (N46K, N→K), 62 (L→F, L62F), 93 (A93V, A→V),132 (S→L, S132L), 134 (I→T, I134T), 136 (H136Y, H→Y), 290 (I290M, I→M), and 323 (I323V, I→V), and the insertion of a single amino acid, Aspartate, in position 55 (D55ins), were detected in the SPPV NISKHI in contrast to the GTPV strains (Table S2, Figure S2). Notably, no changes in positions 46 (N46N) and 98 (A98A) were revealed in the P32 amino acid sequences of the SPPV NISKHI strain and 6/12 GTPV strains. As shown in the dendrogram (Figure 5), the nucleotide sequences of the P32 that were derived from the CapPV representatives clustered strongly in three different branches related to LSDV, GTPV, and SPPV strains, respectively. However, the marked discrimination could be easily observed inside each of the branches, especially in both LSDV and GTPV groups. Less difference was seen between the SPPV NISKNI and other SPPV isolates. Reconstruction of the extended phylogenetic tree that was based on the nucleotide sequences of P32 from whole genomes of widely available CapPV isolates demonstrated a similar observation and also showed a marked polymorphism of this protein (Figure S3).

Comparison of the P32 nucleotide sequence by BLAST analysis (https://blast.ncbi.nlm.nih.gov/Blast.cgi, accessed on 8 August 2025) revealed 100% identity with only several relevant sequences of CapPV strains (n = 122) that are available in the NCBI GenBank (https://www.ncbi.nlm.nih.gov/, accessed on 8 August 2025), which were isolated widely from the target animals, cattle and small ruminants, in different parts of the world during the twentieth and twenty-first centuries (Table S1). In fact, a 100% identity between the P32 of the reference LSDV Neethling_vaccine_LW_1959 was observed with only several relevant sequences of LSDV strains—19/122 (15.6%). The relevant sequences from the remainder of the LSDV strains (66/122, 54.1%) demonstrated an identity of no more than 99–99.9%. The SPPV vaccine strain NISKHI, similarly to other SPPV (32/122, 26.2%) and GTPV (12/122, 9.8%) strains (overall 44/122, 36.10%), possessed P32 relevant sequences with a lower identity— about 97.7–97.9%. The revealed polymorphism has also affected the P32 protein B- and T-cell epitope formation as was predicted by the *in silico* method (see Table 2, Table 3 and Table 4).

### 3.6. Epitope Analysis of P32 Protein by In Silico Method

#### 3.6.1. B-Cell Linear Epitopes

Overall, a similar number (n = 8) of potential linear B-cell epitopes were predicted for the P32 sequences from the SPPV vaccine, NISKHI, as well as for the LSDV Neethling vaccine. However, only half of them (4/8, 50%) were identical in both sequences (Table 2). Thus, the epitopes 3, 4, 6, and 8 had an identical length in the range of 5–27 amino acid residues in both P32 protein sequences, while the positions of each of them differed slightly, with a right shift by 1–2 steps in the SPPV NISKHI. The pairwise comparison demonstrated a certain difference in the epitopes 1, 2, 5, and 7, namely one to four additional amino acids in their compositions in P32 from either the SPPV vaccine NISKHI or the LSDV vaccine Neethling. No epitopes possessed both allergenic and toxic activity according to the predictive analysis. However, P32 of the LSDV vaccine Neethling had four epitopes (the epitopes 1, 2, 4, and 5) with antigenic activity, while P32 from the SPPV vaccine NISKHI—only three (epitopes 1, 2, and 4).

#### 3.6.2. B-Cell Conformational Epitopes

Marked differences were revealed in the number and compositions of the discontinuous or conformational B-cell epitopes predicted for the P32 protein from either the SPPV NISKHI or the LSDV Neethling. In fact, nine epitopes were found in the relevant protein sequence of P32 from the SPPV NISKHI, while only four epitopes were discovered in the one from the LSDV Neethling (Table 3). Moreover, no epitopes of the P32 protein were identical to those of each of the strains. No individual residues involved in the formation of the epitopes 2 and 4 in the LSDV Neethling were identified in any epitopes of the SPPV NISKHI, and vice versa—no individual residues found in the epitopes 2, 5, 6, and 8 of the SPPV NISKHI were present in the LSDV Neethling. Nevertheless, some individual residues were identified in the P32 protein derived from both the LSDV and SPPV strains. Thus, one of the four residues involved in the formation of epitope 1 in the SPPV NISKHI was also a part of the epitope 1 (1/67) of the LSDV Neethling. Almost half of the residues (15/32) of epitope 3 in the SPPV NISKHI were revealed in the composition of epitope 1 in the LSDV Neethling. Only 4/34 individual residues of epitope 4 in the SPPV NISKHI were also identified in epitope 1 (4/67) of the LSDV Neethling. Finally, only one individual residue, which was presented in either epitope 7 (1/7) or epitope 9 (1/13) in the SPPV NISKHI, was involved in the formation of epitopes 1 (1/67) and 3 (1/45) in the LSDV Neethling.

The pairwise models for 3D structures of the individual predicted immunoreactive protein linear (Table 2) and conformational epitopes (Table 3) for P32 from each of the strains, the LSDV Neethling and the SPPV NISKHI, demonstrated a surface location that was available for interaction with the homologous specific antibodies. However, a certain space configuration could be changed (Figures S4 and S5).

#### 3.6.3. T-Cell Epitopes

In total, five T-cell epitopes were predicted for the P32 protein sequence of each of the strains, either the SPPV NISKHI or the LSDV Neethling. Notably, four out of five of these epitopes were identical for P32 from both strains, and the fifth one had only a single substitution in the last amino acid. All the epitopes possessed predicted antigenic and immunogenic activity and were non-allergenic (Table 4).

Summarizing the data obtained by the *in silico* predictions for both B-cell and T-cell epitopes of the P32 protein, we can see an overlap of some of the relevant sequences (Figure S6). Thus, all five T-cell epitope compositions were revealed inside the B-cell sequences that were related to the epitopes: 1 (T-cell epitopes 2–4), 2 (T-cell epitope 5), and 5 (T-cell epitope 1). Similar data were obtained for the P32 protein from both strains, the LSDV Neethling (Figure S6a) and the SPPV NISKHI (Figure S6b).

## 4. Discussion

In this study, we investigated the specificity and duration of the humoral immune response in cattle before and after vaccination and revaccination with a live SPPV-based vaccine against LSD in the course of 15 months. For this purpose, samples of sera and antisera were collected in the territories of the Saratov region, in which no LSD cases and outbreaks were registered after 2019 [13]. Additionally, since 2017, in this region, which borders another country (the Republic of Kazakhstan), target animals have been annually immunized with a live SPPV-based vaccine [17]. The same animals were tested in each of the groups, either group A (before the first immunization at the age of 6 months, one year later before revaccination, after the first vaccination and revaccination, respectively) or group B, which had not yet been subjected to the vaccination campaign (before and after the first vaccination and subsequent revaccination, as well).

Recently, the humoral immune response after the vaccination and revaccination of cattle with homologous vaccines against LSD was carefully investigated by different immunoserological techniques such as the virus neutralization test (VNT), indirect fluorescent antibody test (IFAT), Western blot, agar gel immunodiffusion, and ELISA [36,37,38,60,61,62,63,64]. The commercially available Capripox double-antigen multi-species ELISA that is based on the recombinant CapPV structural protein P32 showed more sensitivity and was more useful for the detection of antibodies to CapPV compared with VNT and IFAT [36,37,38,46]. Therefore, initially in our research, we used this ELISA for the evaluation of the humoral immune response in cattle that were vaccinated against LSD with a heterologous vaccine. However, only a secondary immune response to the SPPV-based vaccine in revaccinated animals could be detected by ELISA. In fact, this test was absolutely uninformative in all other sampling time points, before vaccination and revaccination, as well as after the first vaccination (Table 1). Our findings are in good correlation with the recent observation that was reported by Haegeman et al. [65] in which only one of seven target animals vaccinated with the homologous LSD vaccine was seropositive when using the same commercial IDvet-ELISA on day 16 post-vaccination, while five of seven animals demonstrated seroconversions only just after a booster in the form of an experimental challenge with the wild-type LSDV strain. Additionally, the remainder of the seven immunized animals remained seronegative in IDvet-ELISA even after the booster injection. Similar results were obtained recently by Philips et al. [66]. Overall, based on the data obtained, we can conclude that it was difficult to assess the humoral immune response that was induced by the SPPV-based vaccine using IDvet-ELISA. However, the seroconversion in cattle following the first immunization with homologous LSDV-based vaccines was detectable just after 2 weeks post-inoculation until up to 10 months in individual animals, as reported [36,37,38,61,62,63]. Nevertheless, SPPV-based vaccines are successfully used in different countries, resulting in a decrease in the number of LSD outbreaks. In Russia during the last years, only sporadic cases were registered in a few regions, mainly in Eastern Siberia [13]. The immunogenicity of SPPV-based vaccines and their ability to induce a high level of population immunity against LSD at 1 month in more than 80% of vaccinated cattle have been recently reported [35]. Moreover, the antibody response of heterologous sera to SPPV and LSDV may differ, highlighting a more pronounced immune response induced by the homologous vaccines compared to the heterologous ones [29,39]. Therefore, we additionally analyzed each of the sera by immunoblotting. As expected, this technique was very useful to define the number of seropositive animals (Table 1) and to detect immunoreactive proteins at each of the sampling time points in group A (Figure 3) and in the majority of them in group B (Figure 4).

In fact, the SPPV-based vaccine was able to induce a humoral immune response in 100% of the vaccinated and 100% of the revaccinated animals aged 6–21 months in group A (Table 1). Moreover, the number of seropositive animals in this group was more than 80% one year after the first vaccination. Importantly, we could identify at least three immunodominant proteins, P34, P44, and P66, which were detectable in the antisera of the majority of the animals (more than 50–76%) after the first vaccination and after revaccination, as well. Additionally, we found two other proteins, P24 and P56, interacting with serum antibodies in about half of vaccinated and revaccinated animals. Other proteins were also immunoreactive, although in a smaller number of animals (Figure 3). Using the serum from a cow with LSD, we also identified 7 cross-reactive proteins in the SPPV, among which there were immunodominant ones, P56 and P66 (Figure 3). From our point of view, these data could probably favor the involvement of these proteins in the development of protective immunity against LSD.

Another interesting finding was the presence of serum antibodies in 6-month-old naive calves unvaccinated with any vaccines, including the SPPV-based vaccine (Table 1). In fact, when the blood samples were taken for the first time, all of these animals (group A) were suckling calves from beef farms. Due to a 100% vaccination rate with SPPV-based vaccines in the territory of the Saratov region in the last several years, each cow or heifer received 2–3 or more doses of the vaccine, which was likely to have provoked a specific humoral immune response in the vaccinees. According to several reports [34,36,61], passive antibody transfer from seropositive mothers vaccinated with the LSDV-based vaccine to their newborn calves could be registered 14 days after their birth. These observations are in absolute correlation with our data, providing a good explanation of the detection of maternally derived serum antibodies belonging to the SPPV-based vaccine in calves with no lifetime intravital immunizations. The absence of any specific antibodies to the SPPV-based vaccine-related proteins in the sera of naive adult cattle in group B before the first immunization (Table 1) certainly supports this hypothesis. Overall, this finding may be considered as another argument in favor of the positive effect of regular vaccination with SPPV-based vaccines for both adult animals and calves to provide protection against LSD. We identified seven immunoreactive proteins, P24, P34, P40, P42, P44, P56, and P66—the homologous antibodies to which were presented in the sera of 40–50% calves of group A before their active lifetime immunization with the SPPV-based vaccine. However, these observations require stronger evidence from separate, extended research.

A further important finding of this study was the negative correlation between the age of revaccinated cattle and the number of antibody seropositive responses in ELISA (Figure 2). According to the data obtained, the seroconversion rate was significantly higher in young animals compared with adult cattle (Figure 1), while no difference in the blot’s band breadths, which could be associated with cattle of different ages, was observed. However, both groups of animals demonstrated a similar secondary immune response to the SPPV-based vaccine after revaccination, while the spectrum of immunoreactive proteins strongly differed in each of the groups investigated by means of the immunoblotting technique. A lower number of immunoreactive proteins (n = 19) were recognized by the antisera of the young cattle aged 6–21 months (Figure 3) compared with those which gave positive reactions (n = 25) with the antisera from adult animals (Figure 4) at the age of 31 months and older; with a median age of 60.5 months (31.0–86.0) (Table 1). Importantly, nine proteins were detectable in the antisera of only revaccinated adult cattle in group B, while no positive reactions to the same proteins were registered in the antisera of the young animals in group A (Figure 4). Furthermore, at least eight proteins were regularly involved in the seroconversion of the animals from both the A and B groups, indicating probable similar mechanisms of the humoral immune response induced by the SPPV-based vaccine in cattle regardless of age. Overall, these data clearly show a certain difference in the specificity of the secondary humoral immune response in cattle depending on the immunization route and livestock age for the primary inoculation with the SPPV-based vaccine, as well as the number of vaccinations. If the immune system weakens in older animals, similarly to the majority of mammalians, the question is whether they require larger immunizing doses and/or a greater number of vaccine injections together with novel immunomodulators and adjuvants in order to induce prompt and stronger seroconversion or not. As such, this will be the goal of our next investigation.

To clarify the negative responses seen in the P32-based commercial ELISA, we hypothesized a possible polymorphism in this protein, which was considered to be identical for three CapPV representatives, LSDV, SPPV, and GTPV [45]. In fact, this protein demonstrated a marked polymorphism that is typical for each of the CapPV species (Table S2, Figure S2), which was supported by phylogenetic analysis using 122 different CapPV strains (Figure S3). However, P32 also showed a marked intraspecies polymorphism (Table S1).

Further analysis revealed significant differences in the number and compositions of both linear and conformational B-cell epitopes of P32 in the SPPV and LSDV vaccine strains (Table 2, Table 3 and Table 4, Figure S6), providing great changes in the relevant epitope space configuration (Figures S4 and S5). The additional question is whether the folding of the recombinant P32 used in the commercial ELISA was correct and related to its unique native, three-dimensional structure. Could spontaneous folding interrupt the interaction of P32-specific serum antibodies presented in seropositive cattle to the immunoreactive epitopes of this protein? This may certainly explain the positive responses in this ELISA, mainly after booster injections of the vaccine, but not in the period between vaccination and revaccinations. In contrast to ELISA, in immunoblotting, vaccine-induced antibodies could directly interact with the immunoreactive epitopes of the monomeric form of the native protein P32, demonstrating the contribution of this protein to the seroconversion of cattle immunized with the SPPV-based vaccine. Taken together, these observations may explain the absence of detectable positive reactions in ELISA with P32 protein (Table 1) and certainly may correlate and support a difference in the humoral immune response to SPPV and LSDV that was recently reported by Berguido et al. [39]. Moreover, the less pronounced serological response to the SPPV-based vaccine, compared to homologous and even GTPV-based vaccines [29,67], should be taken into consideration. However, the immune response elicited due to the SPPV-based vaccine could be detected by the IDvet-ELISA after revaccination as a booster to provoke an enhanced antibody response to P32 antigen.

Several limitations of our study should be specified. First of all, a virus neutralization test was not performed, which raised doubts about assigning the antibodies identified by immunoblotting as functional ones. Secondly, our study lacks the direct evidence of antibody transfer from vaccinated dams to their unvaccinated calves due to the absence of the dam sera and colostrum samples. Also, the identification of the immunodominant proteins was done based on molecular weight only, making it unavailable to precisely recognize each of them as previously known SPPV antigens. Finally, more complex statistical analysis, which could assess the influence of different additional factors and random effects, must be fully fed to draw a conclusion between the age and seroconversion rates in cattle vaccinated with the SPPV vaccine.

## 5. Conclusions

In conclusion, annual vaccination of cattle with the live heterologous SPPV-based vaccine was able to induce seroconversion in the majority (up to 80–100%) of the target animals. However, a certain negative correlation between the age of revaccinated cattle and the number of antibody seropositive responses in immunoserological assays was found. The marked polymorphism of P32, detected in each of the CapPV representatives, LSDV, SPPV, and GTPV, could reduce the diagnostic value of this protein, substantiating the need to search for alternative effective markers of humoral immune response in animals vaccinated against LSD. The number of proteins detected by immunoblotting could be potentially promising immunoserological markers of seroconversion in both naive young calves and adult animals before and post-vaccination, as well as revaccination.

## Figures and Tables

**Table 1:** Comparative number of seropositive cattle in IDvet-ELISA and immunoblotting.

Group	Sup-Group	The Time Points of Sampling	Age, Months, Median (25th–75th Percentile)	The Number of Positive Responses in					
				IDvet-ELISA		Immunoblotting		PCR	
				Abs.	%	Abs.	%	Abs.	%
**A**	1.	Before the first vaccination	6.0	0/30	0	24/30	80.0	0/30	0
	2.	28 days post-vaccination	7.0	0/30	0	30/30	100	0/30	0
	3.	One year post-vaccination, one month before revaccination	17.0	0/30	0	25/30	83.3	0/30	0
	4.	28 days post-revaccination	19.0	25/30	83.3	30/30	100	0/30	0
	5.	Three months post-revaccination	21.0	0/30	0	N/A	N/A	0/30	0
**B**	6.	Before the first vaccination	46.5 (17.0–72.0)	0/30	0	0/30	0	0/30	0
	7.	28 days post-vaccination	47.5 (18.0–73.0)	0/30	0	0/30	0	0/30	0
	8.	One year post-vaccination, before revaccination	59.5 (30.0–85.0)	0/30	0	0/30	0	0/30	0
	9.	28 days post-revaccination	60.5 (31.0–86.0)	9/30	30	30/30	100	0/30	0
	10.	Three months post-revaccination	62.5 (33.0–88.0)	0/30	0	30/30	100	0/30	0
**C**	11.	Unvaccinated with no LSD (negative control)	6.0	0/3	0	0/3	0	0/3	0
	12.	Vaccinated with LSD (positive control)	>6.0	1/1	100	1/1	100	1/1	100

N/A: Not available—due to the insufficient sample volume.

**Table 2:** Linear B-cell epitopes of P32 derived from either the SPPV vaccine strain NISKHI or the LSDV Neethling_vaccine_LW_1959 that were predicted *in silico*.

CapPV Strain ID	Epitope	B-Cell Epitope Peptide Composition	Position, Start–end	Number of Residues	Immunogenicity		Antigenicity		Allergenicity	Toxicity	3D Structure
					Scores	Status	Scores	Status			
The LSDV Neethling LW1959	1	IVGREISDVVPELKSD	11–**26**	**16**	0.02868	Immunogen	0.7722	Antigen	Non-Allergen	None	Figure S4—1
	2	KVDTVKDFKNSDVNFF**F**KDKKDISLS	**33–58**	**26**	−0.57546	None	1.0605	Antigen	Non-Allergen	None	Figure S4—2
	3	**VEKSGGVENFTEYFSGLCNALCTKEAK**	**67–93**	27	0.03989	Immunogen	0.3237	Non-Antigen	Non-Allergen	None	Figure S4—3
	4	**DIKNSEN**	**110–116**	7	−0.14336	None	1.5066	Antigen	Non-Allergen	None	Figure S4—4
	5	**IEMQ**EKNI	**139–146**	**8**	−0.28452	None	2.1634	Antigen	Non-Allergen	None	Figure S4—5
	6	**TFHNSNSRILFNQENNNFMYSYTGGYD**	**154–180**	27	−0.21097	None	0.3513	Non-Antigen	Non-Allergen	None	Figure S4—6
	7	**N**EIIKNKGISTS	**198–209**	**12**	−0.20784	None	0.0808	Non-Antigen	Non-Allergen	None	Figure S4—7
	8	**KELKL**	**219–223**	5	−0.2206	None	N/A	N/A	Non-Allergen	None	Figure S4—8
The SPPV NISKHI	1	IVGREISDVVPELKSD**N**	11–**27**	**17**	−0.02268	None	0.8316	Antigen	Non-Allergen	None	Figure S4—1
	2	**YK**KVDTVKDFKNSDVNFF**L**KDKKD**D**ISLS	**31–59**	**29**	−0.69398	None	1.0220	Antigen	Non-Allergen	None	Figure S4—2
	3	**VEKSGGVENFTEYFSGLCNALCTKEAK**	**68–94**	27	0.03989	Immunogen	0.3237	Non-Antigen	Non-Allergen	None	Figure S4—3
	4	**DIKNSEN**	**111–117**	7	−0.14336	None	1.5066	Antigen	Non-Allergen	None	Figure S4—4
	5	EKNI	**144–147**	**4**	−0.0021	None	N/A	N/A	Non-Allergen	None	Figure S4—5
	6	**TFHNSNSRILFNQENNNFMYSYTGGYD**	**155–181**	27	−0.21097	None	0.3513	Non-Antigen	Non-Allergen	None	Figure S4—6
	7	EIIKNKGISTS	**200–210**	**11**	−0.34876	None	0.2495	Non-Antigen	Non-Allergen	None	Figure S4—7
	8	**KELKL**	**220–224**	5	−0.2206	None	N/A	Not Available	Non-Allergen	None	Figure S4—8

The identical individual residues involved in the formation of the relevant epitopes of P32 protein in both the SPPV NISKHI and the LSDV Neethling are in a bold font; variations in either amino acid sequences or positions, or number of residues are in red; N/A—not available.

**Table 3:** Conformational B-cell epitopes of P32 derived from either the SPPV vaccine strain NISKHI or the LSDV Neethling_vaccine_LW_1959 that were predicted *in silico*.

CapPV Strain ID	Epitope	B-Cell Epitope Residues and Position	Number of Residues	Score
The LSDV Neethling_vaccine_LW_1959	1	*A:N27*, *A:I29*, *A:F30*, A:K33, A:V34, A:D35, A:T36, A:V37, A:K38, A:D39, A:F40, A:K41, A:N42, A:S43, A:D44, A:V45, A:N46, A:F47, A:F48, *A:F49*, *A:K50*, *A:D51*, A:K52, A:D54, *A:I55*, *A:S56*, *A:L57*, *A:S58*, *A:V67*, *A:E68*, *A:K69*, *A:S70*, *A:G71*, A:G72, *A:V73*, *A:E74*, *A:N75*, *A:F76*, *A:T77*, *A:E78*, *A:F80*, *A:S81*, *A:G82*, *A:L83*, *A:C84*, *A:N85*, *A:A86*, *A:L87*, *A:C88*, *A:T89*, *A:K90*, *A:E91*, *A:K93*, A:G178, *A:Y179*, *A:N198*, *A:E199*, *A:I201*, *A:K202*, *A:N203*, *A:K204*, *A:G205*, *A:I206*, *A:S207*, *A:T208*, *A:S209*, *A:F212*	67	0.723
	2	*A:K219*, *A:E220*, *A:L221*, *A:K222*, *A:L223*	5	0.67
	3	*A:K117*, A:D125, *A:T127*, *A:D130*, *A:L131*, *A:I132*, *A:T133*, *A:N136*, *A:I139*, *A:E140*, *A:Q142*, *A:E143*, *A:K144*, *A:F155*, *A:H156*, *A:N157*, *A:S158*, *A:N159*, *A:S160*, *A:R161*, *A:I162*, *A:L163*, *A:N165*, *A:Q166*, *A:E167*, *A:N168*, A:N169, A:N170, *A:F171*, *A:M172*, *A:Y173*, *A:S174*, *A:Y175*, *A:T176*, *A:G177*, *A:N229*, *A:D230*, *A:S231*, A:S232, *A:K233*, *A:Y234*, *A:I235*, *A:L236*, *A:H237*, *A:N238*	45	0.636
	4	*A:A2*, *A:D3*, *A:D110*, *A:K112*, *A:N113*, *A:S114*, *A:E115*, *A:N116*	8	0.573
The SPPV vaccine strain NISKHI	1	A:F47, A:F48, **A:L49**, A:K52	4	0.841
	2	**A:F81**, **A:S82**, **A:G83**, **A:L84**, **A:C85**, **A:N86**, **A:A87**, **A:L88**, **A:C89**, **A:T90**, **A:K91**, **A:E92**, **A:K94**, **A:G179**, **A:Y180**, **A:E200**, **A:K203**, **A:N204**, **A:K205**, **A:G206**, **A:I207**, **A:S208**, **A:T209**, **A:S210**, **A:F213**	25	0.746
	3	**A:Y31**, **A:K32**, A:K33, A:V34, A:D35, A:T36, A:V37, A:K38, A:D39, A:F40, A:K41, A:N42, A:S43, A:D44, A:V45, A:N46, **A:K67**, **A:V68**, **A:E69**, **A:K70**, **A:S71**, A:G72, **A:G73**, **A:V74**, **A:E75**, **A:N76**, **A:F77**, **A:T78**, **A:E79**, **A:S106**, **A:Y107**, **A:I202**	32	0.698
	4	**A:I134**, **A:N137**, **A:I140**, **A:E141**, **A:E144**, **A:K145**, **A:F156**, **A:H157**, **A:N158**, **A:S159**, **A:N160**, **A:S161**, **A:R162**, **A:I163**, **A:L164**, **A:N166**, **A:Q167**, **A:E168**, A:N169, A:N170, **A:N171**, **A:F172**, **A:M173**, **A:Y174**, **A:S175**, **A:Y176**, **A:T177**, A:G178, **A:N230**, **A:D231**, A:S232, **A:S233**, **A:K234**, **A:Y235**	34	0.69
	5	**A:K220**, **A:E221**, **A:L222**, **A:K223**, **A:L224**	5	0.655
	6	**A:D111**, **A:K113**, **A:N114**, **A:S115**, **A:E116**, **A:N117**	6	0.569
	7	**A:K53**, A:D54, **A:D55**, **A:I56**, **A:S57**, **A:L58**, **A:S59**	7	0.553
	8	**A:L23**, **A:S25**, **A:D26**	3	0.553
	9	**A:I11**, **A:V12**, **A:G13**, **A:R14**, **A:E15**, **A:I16**, **A:S17**, **A:D18**, **A:V19**, **A:V20**, **A:P21**, **A:E22**, A:D125	13	0.535

The individual residues involved in the formation of the relevant epitopes of the P32 protein of (i) both the SPPV NISKHI and the LSDV Neethling are colored in red; (ii) exclusively in the SPPV NISKHI (bold font); and (iii) only in the LSDV Neethling, highlighted in italic.

**Table 4:** T-cell epitopes of P32 derived from either the SPPV vaccine strain NISKHI or the LSDV Neethling vaccine that were predicted *in silico*.

Epitope	T-Cell Epitope Peptide Composition	Number of Residues	Immunogenicity		Antigenicity		Allergenicity	Presence in CapPV Strain ID	
			Scores	Status	Scores	Status		The LSDV Neethling Vaccine	The SPPV Vaccine Strain NISKHI
1	**KNIDIFQL**	data	0.20756	Immunogen	0.6259	Antigen	Non-Allergen	+	+
2	**ISDVVPEL**	data	0.163	Immunogen	0.6177	Antigen	Non-Allergen	+	+
3	**EISDVVPEL**	data	0.09682	Immunogen	0.9824	Antigen	Non-Allergen	+	+
4	**REISDVVPEL**	data	0.02653	Immunogen	0.6901	Antigen	Non-Allergen	+	+
5	KNSDVNFF**F**	data	0.16993	Immunogen	1.0919	Antigen	Non-Allergen	+	−
6	KNSDVNFF**L**	data	0.16993	Immunogen	0.6480	Antigen	Non-Allergen	−	+

The identical individual residues involved in the formation of the relevant epitopes of P32 protein in both the SPPV NISKHI and the LSDV Neethling are in bold font; variations in either amino acid sequences or positions, or number of residues are in red.

## Data Availability

The dataset and material are available from the corresponding author on reasonable request. All data either generated or analyzed during this study are included in this published article and its supplementary information files.

## Supplementary Materials

The following supporting information can be downloaded at: https://www.mdpi.com/article/10.3390/vaccines13121221/s1, Figure S1A: The Saratov region settings in which the bovine specimens were collected; Figure S1B: The graphical timeline for sampling in the Saratov region; Figure S2: Multiple sequence alignment of the amino acid (a) and nucleotide (b) sequences of the P32 protein derived from the whole genome sequences of CapPVs (LSDV, SPPV, and GTPV representative strains available at the NCBI database (https://www.ncbi.nlm.nih.gov/, accessed on 8 August 2025); Figure S3: Phylogenetic tree based on the nucleotide sequences of P32 from whole genomes of widely available CapPV isolates; Figure S4: B-cell linear epitope peptide composition of P32 protein in either the LSDV Neethling LW1959 or in the SPPV NISKHI; Figure S5: The pairwise 3D ribbon diagrams modeling B-cell conformational epitopes residues of P32 protein on the basis of the relevant original amino acid sequences of P32 from either the LSDV Neethling or the SPPV NISCHI strains; Figure S6: Positions of the B-cell- and the T-cell epitopes predicted by the *in silico* method ElliPro NetMHC v.3.0 (http://tools.iedb.org/ellipro/, 5 August 2025) on the amino acid sequences of either the SPPV vaccine NISKHI (a) or the LSDV Neethling vaccine (b) strains; Table S1: Intraspecies polymorphism of the P32 nucleotide sequence derived from relevant sequences of representative Capripoxvirus strains, available at the NCBI database (https://www.ncbi.nlm.nih.gov/, accessed on 8 August 2025); Table S2: Comparative alignment of the nucleotide sequences of P32 protein derived from the whole genome sequences of the SPPV vaccine strain NISKHI versus the reference LSDV Neethling_vaccine_LW_1959 and other LSDV representatives.

## References

[1] Tuppurainen E.S.M., Venter E.H., Shisler J.L., Gari G., Mekonnen G.A., Juleff N., Lyons N.A., De Clercq K., Upton C., Bowden T.R. (2017). Review: Capripoxvirus diseases: Current status and opportunities for control. Transbound. Emerg. Dis..

[2] Sprygin A., Pestova Y., Wallace D.B., Tuppurainen E., Kononov A.V. (2019). Transmission of lumpy skin disease virus: A short review. Virus. Res..

[3] Kononov A., Byadovskaya O., Wallace B.D., Prutnikov P., Pestova Y., Kononova S., Nesterov A., Rusaleev V., Lozovoy D., Sprygin A. (2020). Non-vector-borne transmission of lumpy skin disease virus. Sci. Rep..

[4] Nesterov A., Mazloum A., Byadovskaya O., Shumilova I., Schalkwyk A.V., Krotova A., Kirpichenko V., Donnik I., ChvalaIlya I., Sprygin1 A. (2022). Experimentally controlled study indicates that the naturally occurring recombinant vaccine-like lumpy skin disease strain Udmurtiya/2019, detected during freezing winter in northern latitudes, is transmitted via indirect contact. Front. Vet. Sci..

[5] Babiuk S., Bowden T.R., Boyle D.B., Wallace D.B., Kitching R.P. (2008). Capripoxviruses: An emerging worldwide threat to sheep, goats and cattle. Transbound. Emerg. Dis..

[6] Datten B., Chaudhary A.A., Sharma S., Singh L., Rawat K.D., Ashraf M.S., Alneghery L.M., Aladwani M.O., Rudayni H.A., Dayal D. (2023). An Extensive Examination of the Warning Signs, Symptoms, Diagnosis, Available Therapies, and Prognosis for Lumpy Skin Disease. Viruses.

[7] Sprygin A., Artyuchova E., Babin Y., Prutnikov P., Kostrova E., Byadovskaya O., Kononov A. (2018). Epidemiological char-acterization of lumpy skin disease outbreaks in Russia in 2016. Transbound. Emerg. Dis..

[8] Akther M., Akter S.H., Sarker S., Aleri J.W., Annandale H., Abraham S., Uddin J.M. (2023). Global burden of lumpy skin disease, outbreaks, and future challenges. Viruses.

[9] Saltykov Y.V., Kolosova A.A., Feodorova V.A. (2022). Update of lumpy skin disease: Emergence in Asian Part of Eurasia. Acta Vet..

[10] Wilhelm L., Ward M.P. (2023). The spread of lumpy skin disease virus across Southeast Asia: Insights from surveillance. Transbound. Emerg. Dis..

[11] Calistri P., De Clercq K., Gubbins S., Klement E., Stegeman A., Cortiñas A.J., Marojevic D., Antoniou S.E., Broglia A. (2020). Lumpy skin disease epidemiological report IV: Data collection and analysis. EFSA J..

[12] Byadovskaya O., Prutnikov P., Shalina K., Babiuk S., Perevozchikova N., Korennoy F., Chvala I., Kononov A., Sprygin A. (2022). The changing epidemiology of lumpy skin disease in Russia since the first introduction from 2015 to 2020. Transbound. Emerg. Dis..

[13] fsvps.gov.ru. https://fsvps.gov.ru/jepizooticheskaja-situacija/rossija/svodnye-kartograficheskie-dannye-hronologii-neblagopoluchija-v-rf-po-osobo-opasnym-i-jekonomicheski-znachimym-boleznjam-zhivotnyh/.

[14] Sprygin A., Pestova Y., Prutnikov P., Kononov A. (2018). Detection of vaccine-like lumpy skin disease virus in cattle and *Musca domestica L. flies* in an outbreak of lumpy skin disease in Russia in 2017. Transbound. Emerg. Dis..

[15] Kononov A., Byadovskaya O., Kononova S., Yashin R., Zinyakov N., Mischenko V., Perevozchikova N., Sprygin A. (2019). Detection of vaccine-like strains of lumpy skin disease virus in outbreaks in Russia in 2017. Arch. Virol..

[16] Sprygin A., Pestova Y., Bjadovskaya O., Prutnikov P., Zinyakov N., Kononova S., Ruchnova O., Lozovoy D., Chvala I., Kononov A. (2020). Evidence of recombination of vaccine strains of lumpy skin disease virus with field strains, causing disease. PLoS ONE.

[17] Saltykov Y.V., Kolosova A.A., Filonova N.N., Chichkin A.N., Feodorova V.A. (2021). Genetic evidence of multiple introductions of lumpy skin disease virus into Saratov Region, Russia. Pathogens.

[18] Haegeman A., De Leeuw I., Mostin L., Campe W.V., Aerts L., Venter E., Tuppurainen E., Saegerman C., De Clercq K. (2021). Comparative evaluation of lumpy skin disease virus-based live attenuated vaccines. Vaccines.

[19] Tuppurainen E., Dietze K., Wolff J., Bergmann H., Beltran-Alcrudo D., Fahrion A., Lamien C.E., Busch F., Sauter-Louis C., Conraths F.J. (2021). Review: Vaccines and vaccination against lumpy skin disease. Vaccines.

[20] Ben-Gera J., Klement E., Khinich E., Stram Y., Shpigel N.Y. (2015). Comparison of the efficacy of Neethling lumpy skin disease virus and x10RM65 sheep-pox live attenuated vaccines for the prevention of lumpy skin disease—The results of a randomized controlled field study. Vaccine.

[21] Abutarbush S.M., Hananeh W.M., Ramadan W., Al Sheyab O.M., Alnajjar A.R., Al Zoubi I.G., Knowles N.J., Bachanek-Bankowska K., Tuppurainen E.S. (2016). Adverse reactions to field vaccination against lumpy skin disease in Jordan. Transbound. Emerg. Dis..

[22] Agianniotaki E.I., Chaintoutis S.C., Haegeman A., Tasioudi K.E., De Leeuw I., Katsoulos P.D., Sachpatzidis A., De Clercq K., Alexandropoulos T., Polizopoulou Z.S. (2017). Development and validation of a TaqMan probe-based real-time PCR method for the differentiation of wild type lumpy skin disease virus from vaccine virus strains. J. Virol. Methods.

[23] Agianniotaki E.I., Tasioudi K.E., Chaintoutis S.C., Iliadou P., Mangana-Vougiouka O., Kirtzalidou A., Alexandropoulos T., Sachpatzidis A., Plevraki E., Dovas C.I. (2017). Lumpy skin disease outbreaks in Greece during 2015–16, implementation of emergency immunization and genetic differentiation between field isolates and vaccine virus strains. Vet. microbiol..

[24] Katsoulos P.D., Chaintoutis S.C., Dovas C.I., Polizopoulou Z.S., Brellou G.D., Agianniotaki E.I., Tasioudi K.E., Chondrokouki E., Papadopoulos O., Karatzias H. (2018). Investigation on the incidence of adverse reactions, viraemia and haematological changes following field immunization of cattle using a live attenuated vaccine against lumpy skin disease. Transbound. Emerg. Dis..

[25] Bamouh Z., Hamdi J., Fellahi S., Khayi S., Jazouli M., Tadlaoui K.O., Fihri O.F., Tuppurainen E., Elharrak M. (2021). Investigation of post vaccination reactions of two live attenuated vaccines against lumpy skin disease of cattle. Vaccines.

[26] Tuppurainen E.S., Pearson C.R., Bachanek-Bankowska K., Knowles N.J., Amareen S., Frost L., Henstock M.R., Lamien C.E., Diallo A., Mertens P.P. (2014). Characterization of sheep pox virus vaccine for cattle against lumpy skin disease virus. Antiviral. Res..

[27] Şevik M., Doğan M. (2017). Epidemiological and molecular studies on lumpy skin disease outbreaks in Turkey during 2014–2015. Transbound. Emerg. Dis..

[28] Tuppurainen E.S., Oura C.A. (2012). Review: Lumpy skin disease: An emerging threat to Europe, the Middle East and Asia. Transbound. Emerg. Dis..

[29] Hamdi J., Bamouh Z., Jazouli M., Boumart Z., Tadlaoui K.O., Fihri O.F., El Harrak M. (2020). Experimental evaluation of the cross-protection between Sheeppox and bovine Lumpy skin vaccines. Sci. Rep..

[30] Gari G., Abie G., Gizaw D., Wubete A., Kidane M., Asgedom H., Bayissa B., Ayelet G., Oura C.A., Roger F. (2015). Evaluation of the safety, immunogenicity and efficacy of three capripoxvirus vaccine strains against lumpy skin disease virus. Vaccine.

[31] Zhugunissov K., Bulatov Y., Orynbayev M., Kutumbetov L., Abduraimov Y., Shayakhmetov Y., Taranov D., Amanova Z., Mambetaliyev M., Absatova Z. (2020). Goatpox virus (G20-LKV) vaccine strain elicits a protective response in cattle against lumpy skin disease at challenge with lumpy skin disease virulent field strain in a comparative study. Vet. Microbiol..

[32] Kumar N., Barua S., Kumar R., Khandelwal N., Kumar A., Verma A., Singh L., Godara B., Chander Y., Kumar G. (2023). Evaluation of the safety, immunogenicity and efficacy of a new live-attenuated lumpy skin disease vaccine in India. Virulence.

[33] Hakobyan V., Sargsyan K., Kharatyan S., Elbakyan H., Sargsyan V., Markosyan T., Vardanyan T., Badalyan M., Achenbach J.E. (2023). The serological response in cattle following administration of a heterologous sheep pox virus strain vaccine for protection from lumpy skin disease; current situation in Armenia. Vet. Sci..

[34] Enul H., Uzar S., Satir E., Sarac F., Adiay C., Parmaksiz A., Colak G., Asar E. (2024). Humoral immune response profile of a cattle herd vaccinated with 5- and 10-times Bakirköy strain sheep pox vaccine under field conditions. Vaccine.

[35] Hakobyan V., Sargsyan K., Elbakyan H., Sargsyan V., Markosyan T., Chobanyan G., Badalyan M., Kharatyan S. (2024). Duration of immunity in cattle to lumpy skin disease utilizing a sheep pox vaccine. Vet. Sci..

[36] Milovanović M., Dietze K., Milićević V., Radojičić S., Valčić M., Moritz T., Hoffmann B. (2019). Humoral immune response to repeated lumpy skin disease virus vaccination and performance of serological tests. BMC Vet. Res..

[37] Krešić N., Šimić I., Bedeković T., Acinger-Rogić Ž., Lojkić I. (2020). Evaluation of serological tests for detection of antibodies against Lumpy Skin Disease Virus. J. Clin. Microbiol..

[38] Milovanović M., Milićević V., Radojičić S., Valčić M., Hoffmann B., Dietze K. (2020). Suitability of individual and bulk milk samples to investigate the humoral immune response to lumpy skin disease vaccination by ELISA. Virol. J..

[39] Berguido F.J., Kangethe R.T., Shell W., Wijewardana V., Grabherr R., Cattoli G., Lamien C.E. (2024). Different neutralizing antibody responses of heterologous sera on sheeppox and lumpy skin disease viruses. Viruses.

[40] Sprygin A., Shalina K., van Schalkwyk A., Mazloum A., Shcherbinin S., Krotova A., Byadovskaya O., Prokhvatilova L., Chvala I. (2023). Molecular and epidemiological analyses of sheeppox outbreaks in Russia from 2013 to 2021. Transbound. Emerg. Dis..

[41] Le Goff C., Lamien C.E., Fakhfakh E., Chadeyras A., Aba-Adulugba E., Libeau G., Tuppurainen E., Wallace D.B., Adam T., Silber R. (2009). Capripoxvirus G-protein-coupled chemokine receptor: A host-range gene suitable for virus animal origin discrimination. J. Gen. Virol..

[42] Ireland D.C., Binepal Y.S. (1998). Improved detection of capripoxvirus in biopsy samples by PCR. J. Virol. Methods.

[43] Feodorova V.A., Zaitsev S.S., Lyapina A.M., Kichemazova N.V., Saltykov Y.V., Khizhnyakova M.A., Evstifeev V.V., Larionova O.S. (2023). Whole genome sequencing characteristics of *Chlamydia psittaci* caprine AMK-16 strain, a promising killed whole cell veterinary vaccine candidate against chlamydia infection. PLoS ONE.

[44] Hughes L., Wilkins K., Goldsmith C.S., Smith S., Hudson P., Patel N., Karem K., Damon I., Li Y., Olson V.A. (2017). A rapid Orthopoxvirus purification protocol suitable for high-containment laboratories. J. Virol. Methods.

[45] Fay P.C., Cook C.G., Wijesiriwardana N., Tore G., Comtet L., Carpentier A., Shih B., Freimanis G., Haga I.R., Beard P.M. (2020). Madin-Darby bovine kidney (MDBK) cells are a suitable cell line for the propagation and study of the bovine poxvirus lumpy skin disease virus. J. Virol. Methods.

[46] Carn V.M., Kitching R.P., Hammond J.M., Chand P. (1994). Use of a recombinant antigen in an indirect ELISA for detecting bovine antibody to capripoxvirus. J. Virol. Methods.

[47] Venkatesan G., Kumar Teli M., Sankar M., Kumar A., Dashprakash M., Arya S., Madhavan A., Ramakrisnan M.A., Pandey A.B. (2018). Expression and evaluation of recombinant P32 protein based ELISA for sero-diagnostic potential of capripox in sheep and goats. Mol. Cell. Probes.

[48] Kushwaha A., Kumar A., Madhavan A., Goswami D., Poulinlu G., Venkatesan G. (2019). Immunogenic proteins of capripox virus: Potential applications in diagnostic/prophylactic developments. Hosts Viruses.

[49] Sumana K., Revanaiah Y., Shivachandra S.B., Mothay D., Apsana R., Saminathan M., Basavaraj S., Reddy G.B.M. (2020). Molecular phylogeny of *Capripoxviruses* based on major immunodominant protein (P32) reveals circulation of host specific sheeppox and goatpox viruses in small ruminants of India. Infect. Genet. Evol..

[50] Tcherepanov V., Ehlers A., Upton C. (2006). Genome Annotation Transfer Utility (GATU): Rapid annotation of viral genomes using a closely related reference genome. BMC Genom..

[51] Tamura K., Stecher G., Kumar S. (2021). MEGA11: Molecular Evolutionary Genetics Analysis Version 11. Mol. Biol. Evol..

[52] Ponomarenko J.V., Bui H.H., Li W., Fusseder N., Bourne P.E., Sette A., Peters B. (2008). ElliPro: A new structure-based tool for the prediction of antibody epitopes. BMC Bioinform..

[53] Feodorova V.A., Lyapina A.M., Khizhnyakova M.A., Zaitsev S.S., Sayapina L.V., Arseneva T.E., Trukhachev A.L., Lebedeva S.A., Telepnev M.V., Ulianova O.V. (2018). Humoral and cellular immune responses to *Yersinia pestis* Pla antigen in humans immunized with live plague vaccine. PLoS Negl. Trop. Dis..

[54] Feodorova V.A., Lyapina A.M., Khizhnyakova M.A., Zaitsev S.S., Saltykov Y.V., Motin V.L. (2020). *Yersinia pestis* antigen F1 but not LcrV induced humoral and cellular immune responses in humans immunized with live plague vaccine—Comparison of immunoinformatic and immunological approaches. Vaccines.

[55] Andreatta M., Nielsen M. (2016). Gapped sequence alignment using artificial neural networks: Application to the MHC class I system. Bioinformatics.

[56] Doytchinova I., Flower D.R. (2007). VAXIJN: A server for prediction of protective antigens, tumour antigens and subunit vaccines. BMC Bioinform..

[57] Calis J.J., Maybeno M., Greenbaum J.A., Weiskopf D., De Silva A.D., Sette A., Keşmir C., Peters B. (2013). Properties of MHC class I presented peptides that enhance immunogenicity. PLoS Comput. Biol..

[58] Gupta S., Kapoor P., Chaudhary K., Gautam A., Kumar R., Raghava G.P., Open Source Drug Discovery Consortium (2013). *In silico* approach for predicting toxicity of peptides and proteins. PLoS ONE.

[59] Nguyen M.N., Krutz N.L., Limviphuvadh V., Lopata A.L., Gerberick G.F., Maurer-Stroh S. (2022). AllerCatPro 2.0: A web server for predicting protein allergenicity potential. Nucleic Acids Res..

[60] Gari G., Biteau-Coroller F., LeGoff C., Caufour P., Roger F. (2008). Evaluation of indirect fluorescent antibody test (IFAT) for the diagnosis and screening of lumpy skin disease using Bayesian method. Vet. Microbiol..

[61] Abdelwahab M.G., Khafagy H.A., Moustafa A.M., Saad M.A. (2016). Evaluation of humoral and cell-mediated immunity of lumpy skin disease vaccine prepared from local strain in calves and its related to maternal immunity. J. Am. Sci..

[62] Norian R., Ahangaran A.N., Vashovi H.R., Azadmehr A. (2016). Evaluation of humoral and cell-mediated immunity of two capripoxvirus vaccine strains against lumpy skin disease virus. Iranian J. Virol..

[63] Varshovi H.R., Norian R., Azadmehr A., Ahangaran A.N. (2018). Immune response characteristics of Capri pox virus vaccines following emergency vaccination of cattle against lumpy skin disease virus. Iranian J. Vet. Sci. Technol..

[64] Regge N. (2024). Lumpy skin disease. Manual of Diagnostic Tests and Vaccines for Terrestrial Animals.

[65] Haegeman A., De Leeuw I., Saduakassova M., Van Campe W., Aerts L., Philips W., Sultanov A., Mostin L., De Clercq K. (2021). The importance of quality control of LSDV live attenuated vaccines for its safe application in the field. Vaccines.

[66] Philips W., Haegeman A., Krešić N., Mostin L., De Regge N. (2024). Neethling Strain-Based Homologous Live Attenuated LSDV Vaccines Provide Protection Against Infection with a Clade 2.5 Recombinant LSDV Strain. Vaccines.

[67] Haegeman A., Philips W., Mostin L., De Leeuw I., Van Campe W., Saegerman C., De Clercq K., De Regge N. (2025). A goatpox but not sheeppox heterologous live attenuated vaccines provide complete protection against lumpy skin disease in cattle under experimental conditions. Sci. Rep..

