# Supplementary material for: Humoral Response in Cattle Vaccinated with the Heterologous Sheeppox Virus Vaccine for Protection Against Lumpy Skin Disease: A Field Study
Source: Vaccines (Basel). 2025 Dec 3;13(12):1221. doi: 10.3390/vaccines13121221 (PMC12737495; doi:10.3390/vaccines13121221)
Supplement: Supplementary file 1 [file vaccines-13-01221-s001.zip › Supplementary Figure S1.pdf]

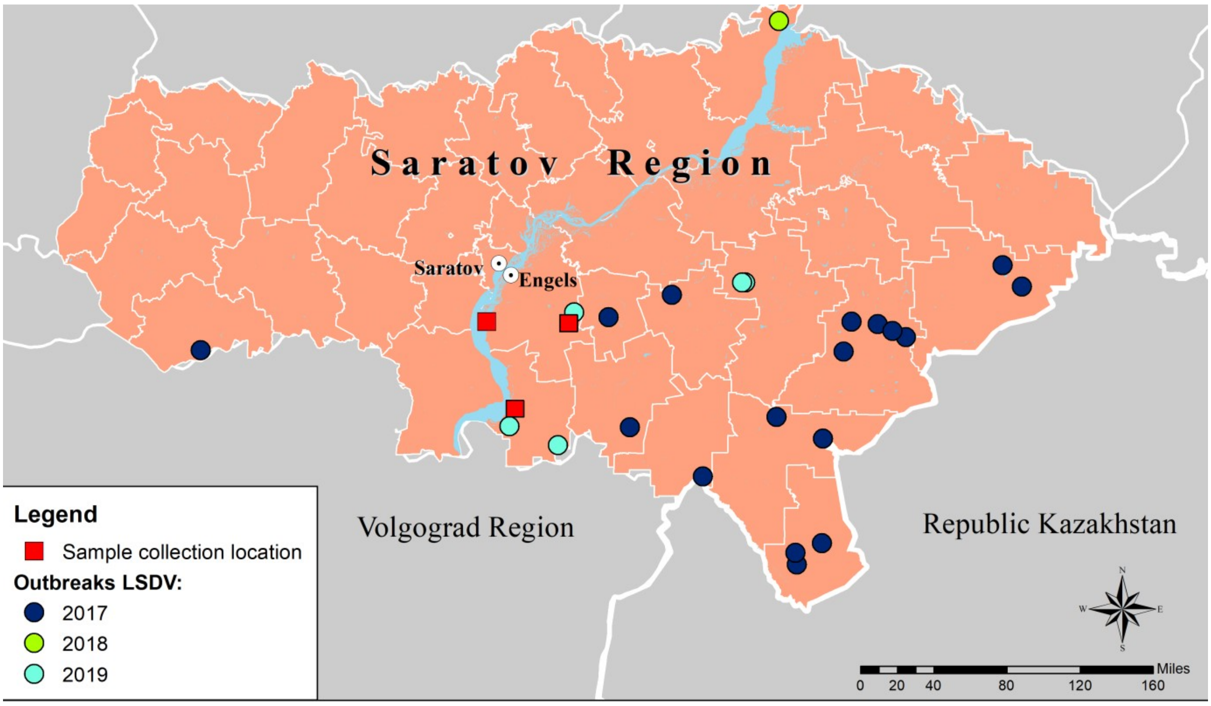

Supplementary Figure S1A. The Saratov Region settings in which the bovine specimens were collected are colored in red squares. The locations of LSD outbreaks on the territory during 2017–2019 are shown as circles.

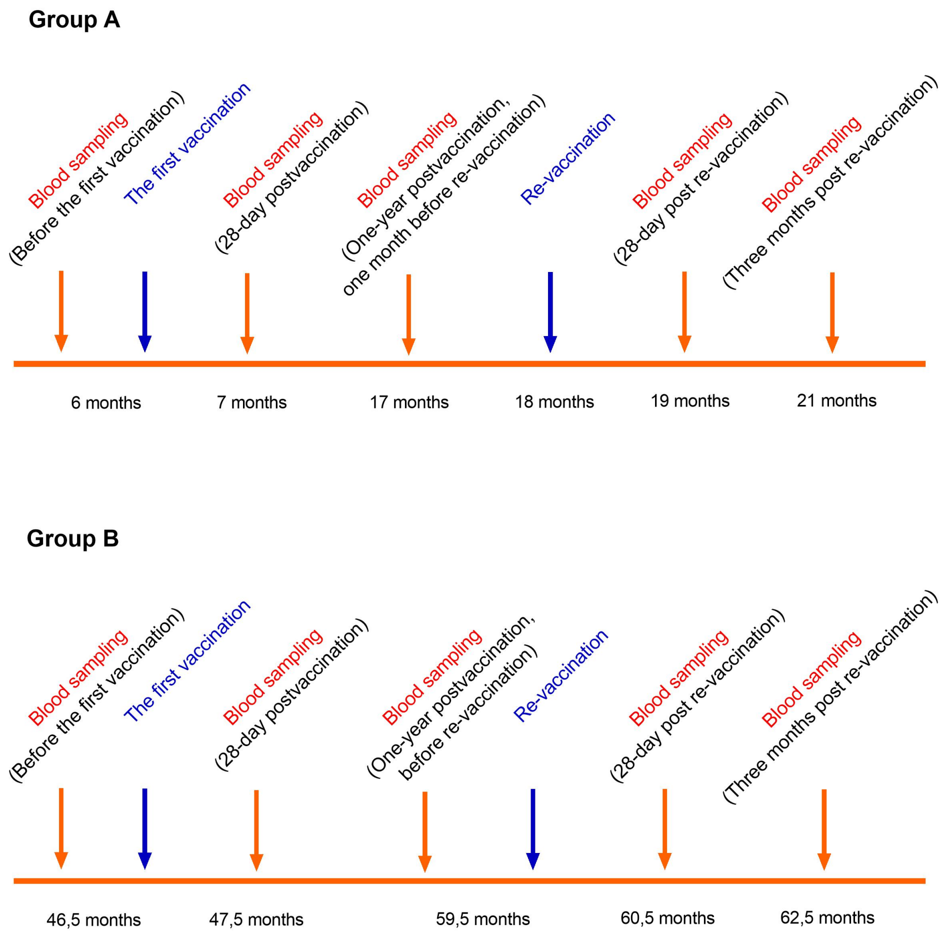

Supplementary Figure S1B. The graphical timeline for sampling in the Saratov Region.
