# Supplementary material for: Humoral Response in Cattle Vaccinated with the Heterologous Sheeppox Virus Vaccine for Protection Against Lumpy Skin Disease: A Field Study
Source: Vaccines (Basel). 2025 Dec 3;13(12):1221. doi: 10.3390/vaccines13121221 (PMC12737495; doi:10.3390/vaccines13121221)
Supplement: Supplementary file 1 [file vaccines-13-01221-s001.zip › Supplementary Figure S2.pdf]

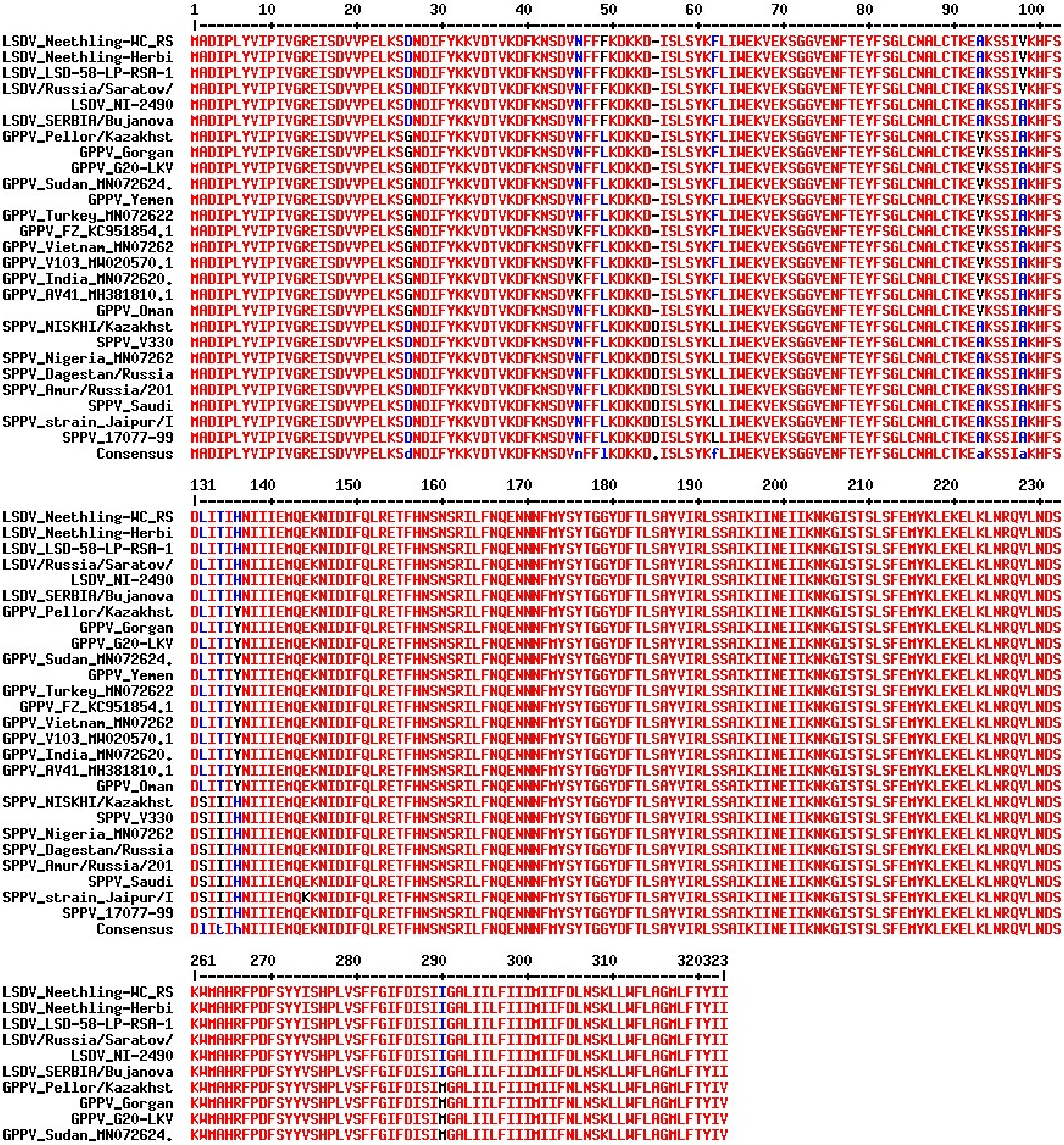

(a)

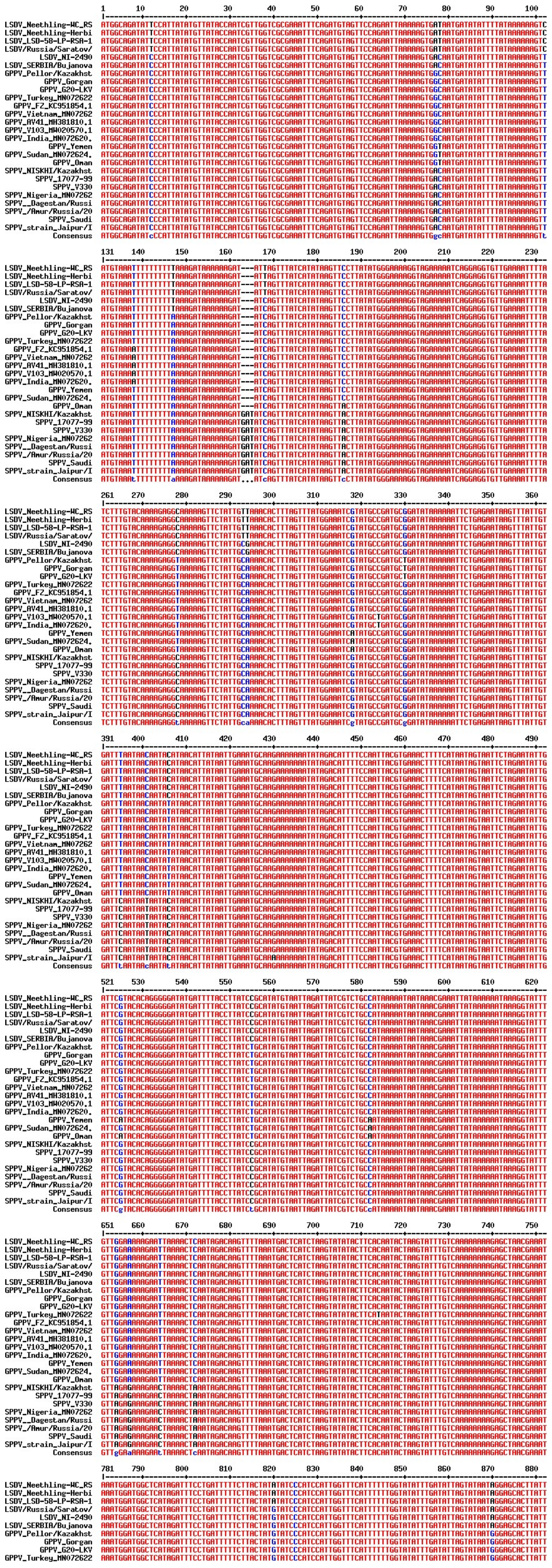

(b)

Supplementary Figure S2. Multiple sequence alignment of the amino acid **(a)** and nucleotide **(b)** sequences of the P32 protein derived from the whole genome sequences of CapPVs (LSDV, SPPV and GTPV representative strains available at the NCBI database (https://www.ncbi.nlm.nih.gov/ (accessed on 26 November 2024)) and presented in Supplementary Table S1.
