# Supplementary material for: Humoral Response in Cattle Vaccinated with the Heterologous Sheeppox Virus Vaccine for Protection Against Lumpy Skin Disease: A Field Study
Source: Vaccines (Basel). 2025 Dec 3;13(12):1221. doi: 10.3390/vaccines13121221 (PMC12737495; doi:10.3390/vaccines13121221)
Supplement: Supplementary file 1 [file vaccines-13-01221-s001.zip › Supplementary Figure S4.pdf]

Epitope #	B-cell linear epitope peptide composition of P32 protein in	
	The LSDV Neethling LW1959	The SPPV NISKHI
1	IVGREISDVVPELKSD	IVGREISDVVPELKSDN
	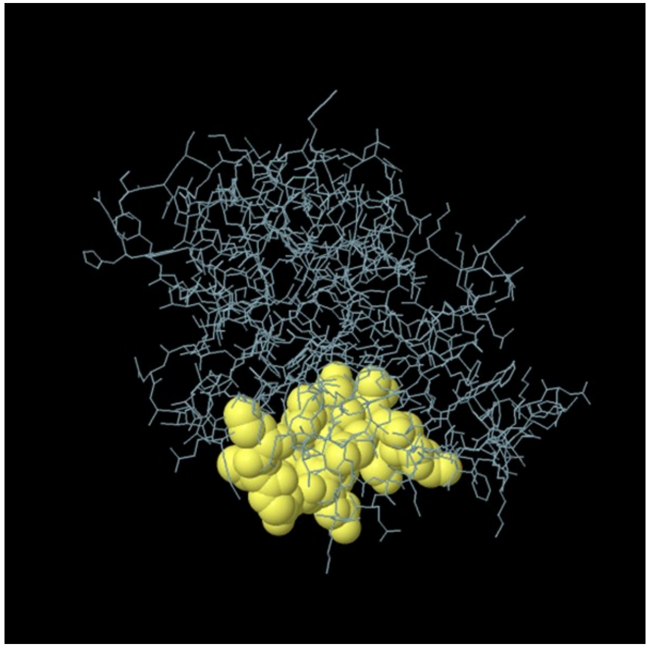	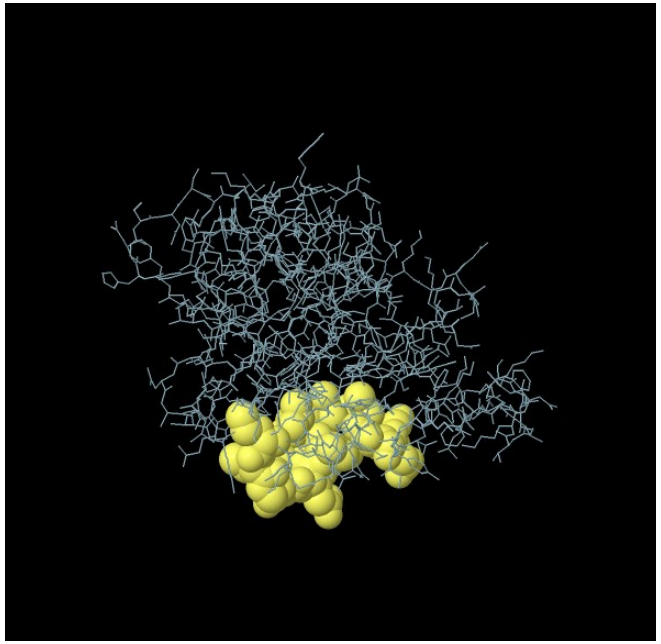
2	KVDTVKDFKNSDVNFFFKDKKDISLS	YKKVDTVKDFKNSDVNFFLKDKKDDISLS

3 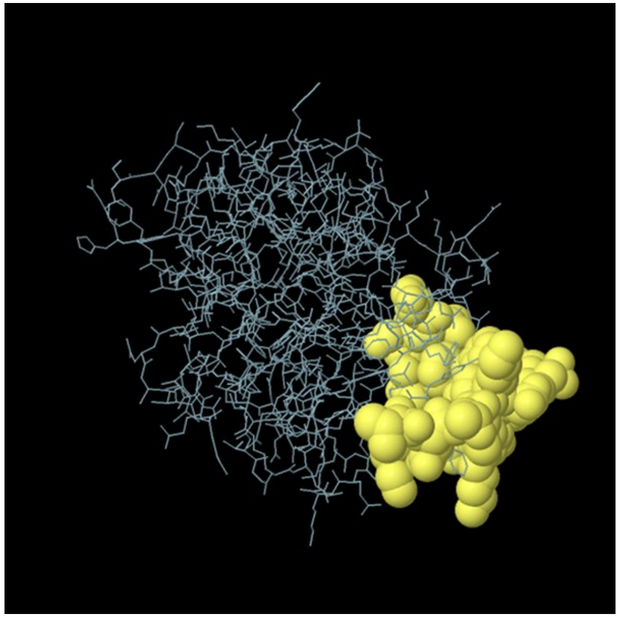

VEKSGGVENFTEYFSGLCNALCTKEAK

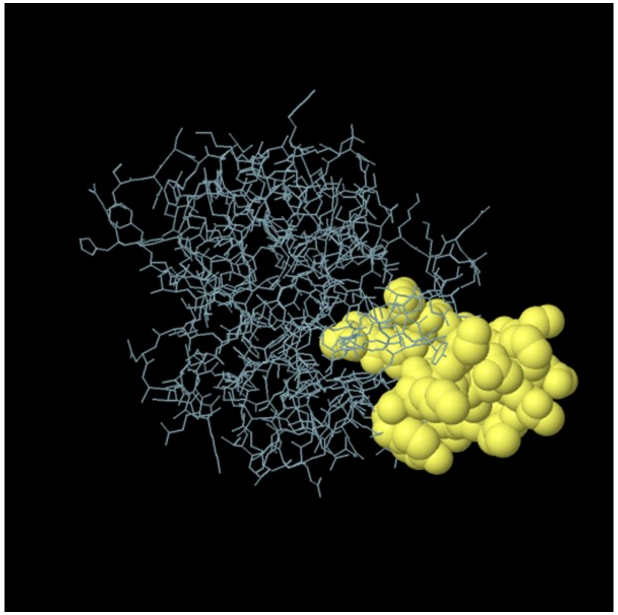

VEKSGGVENFTEYFSGLCNALCTKEAK

4

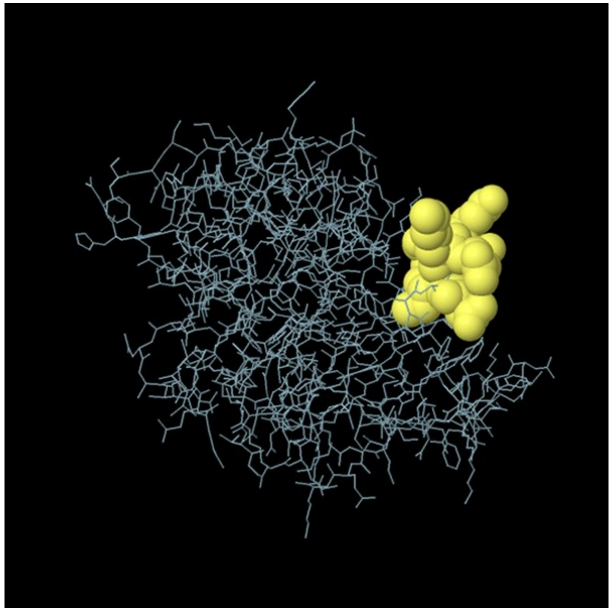

DIKNSEN

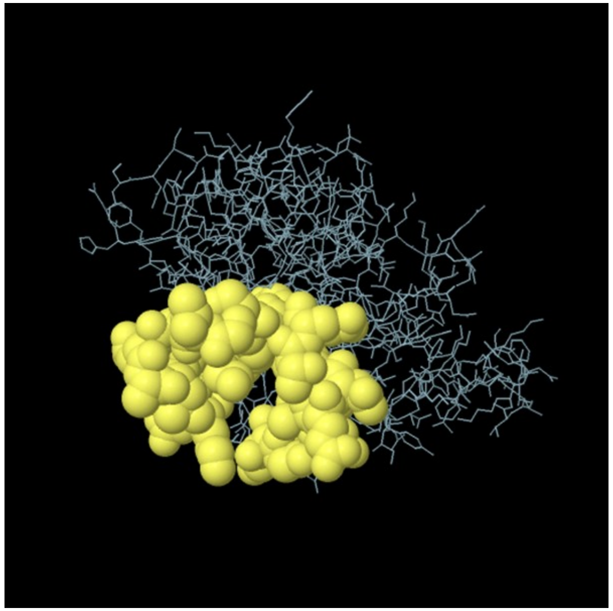

DIKNSEN

5

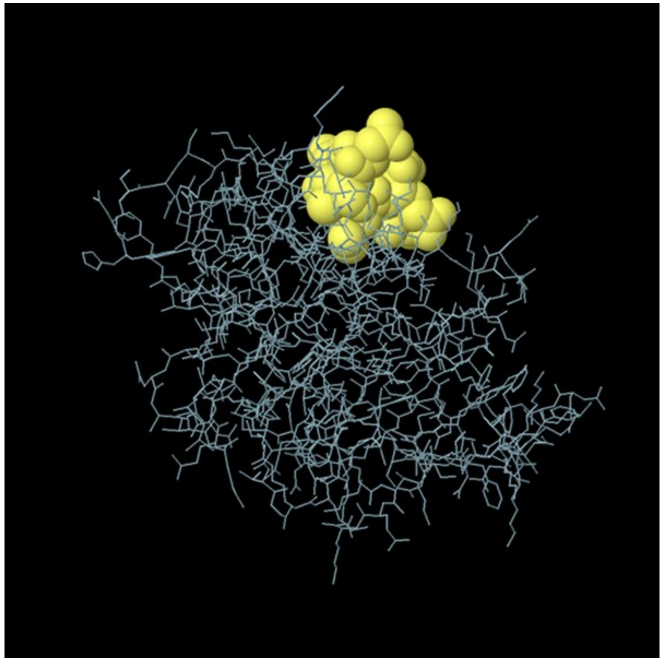

IEMQEKNI

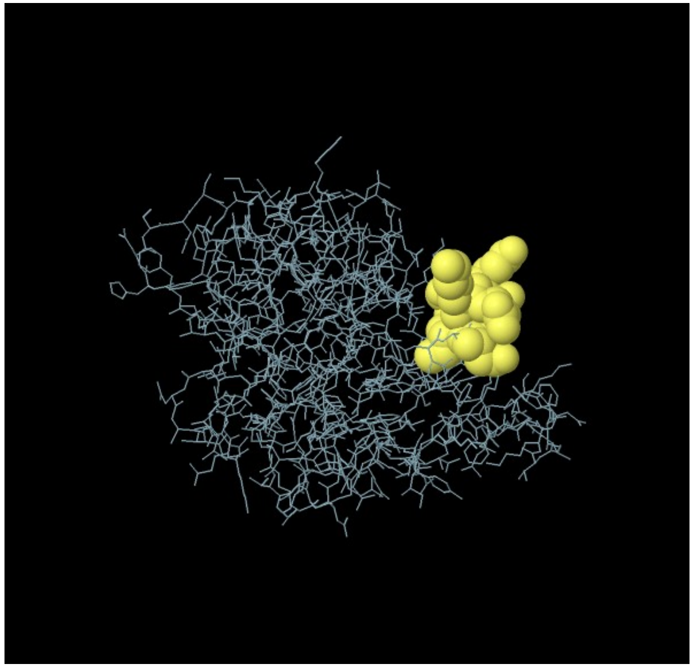

EKNI

6 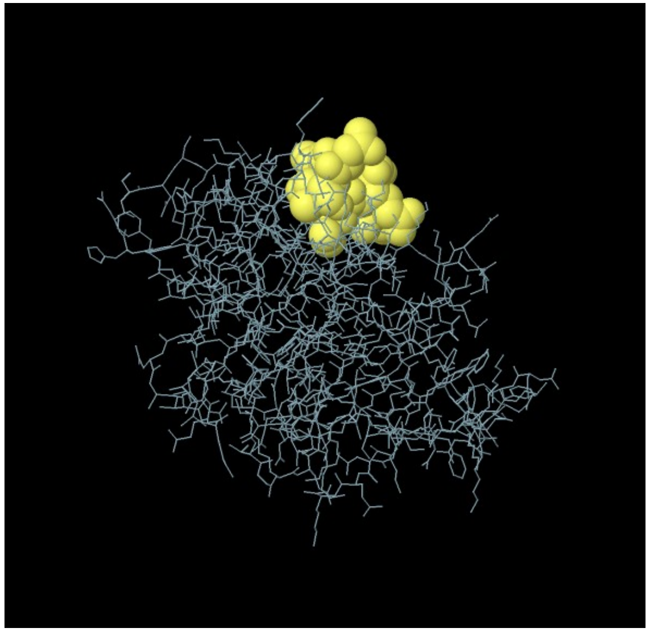 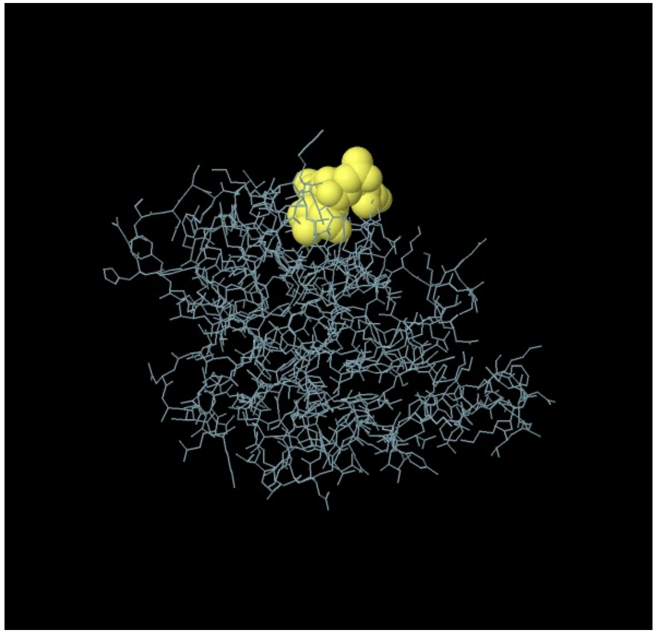

TFHNSNSRILFNQENNNFMYSYTGGYD TFHNSNSRILFNQENNNFMYSYTGGYD

7

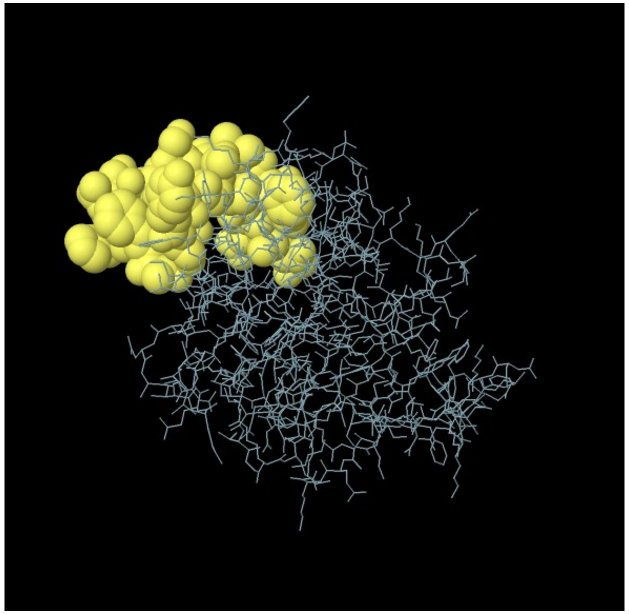

NEIIKNKGISTS

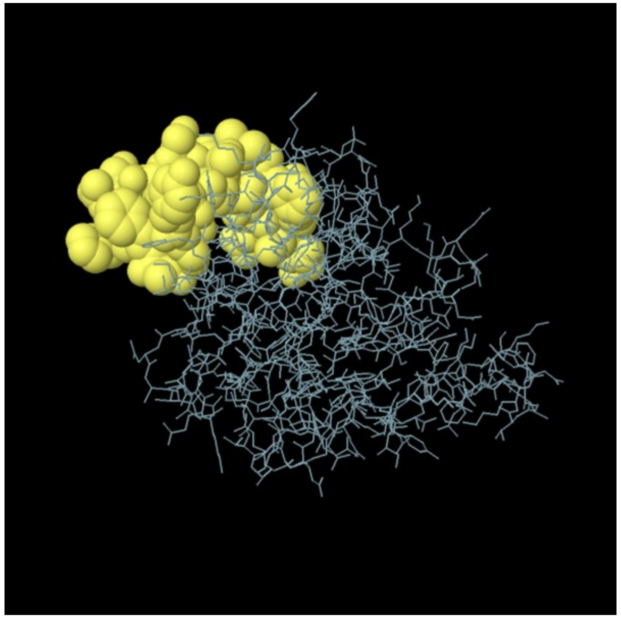

EIIKNKGISTS

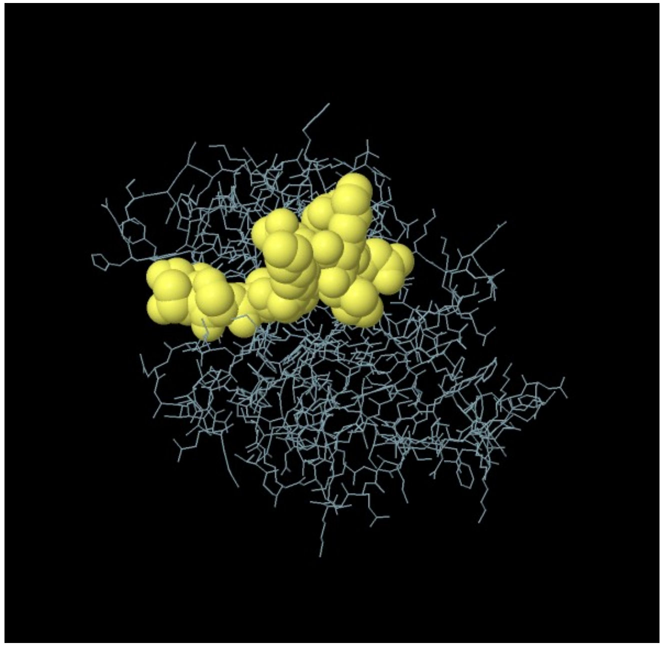

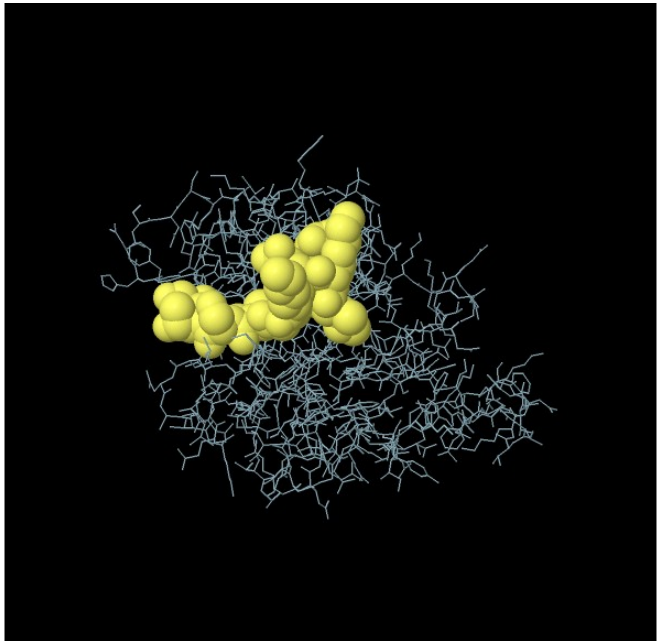

8 KELKL KELKL

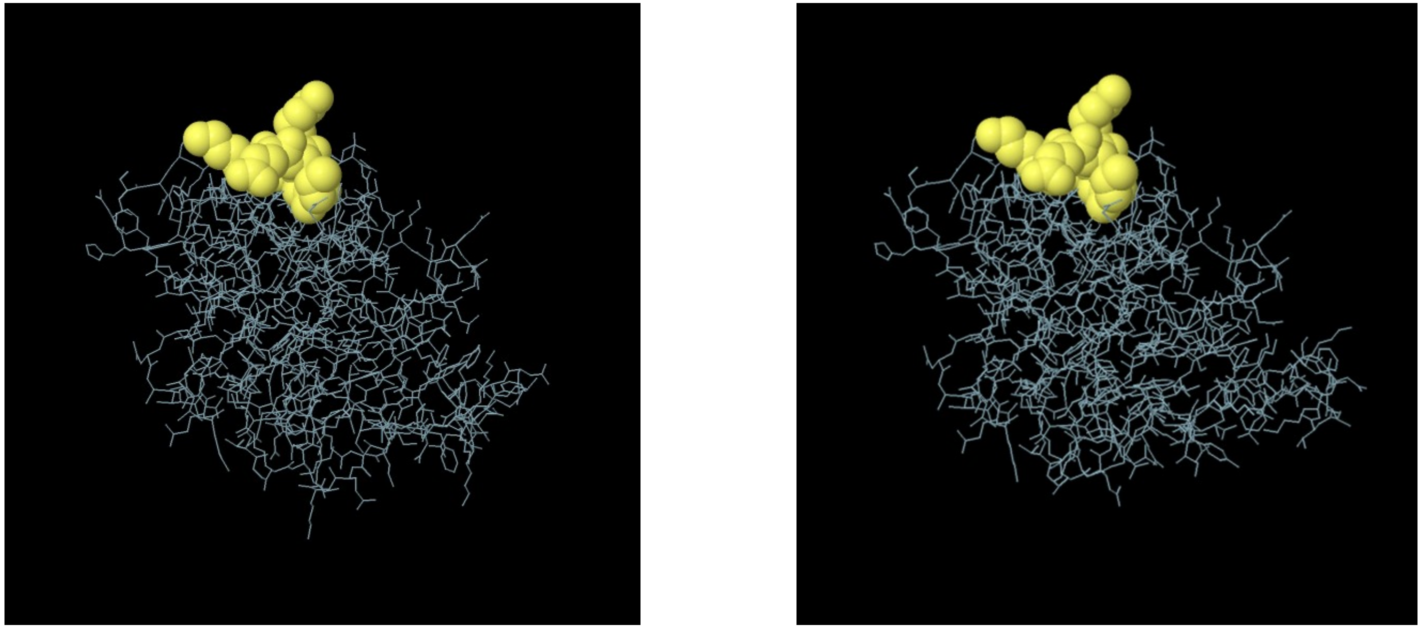

Supplementary Figure S4. B-cell linear epitope peptide composition of P32 protein in either the LSDV Neethling LW1959 or in the SPPV NISKHI. The amino acid substitutions are colored in red.
