# Supplementary material for: Humoral Response in Cattle Vaccinated with the Heterologous Sheeppox Virus Vaccine for Protection Against Lumpy Skin Disease: A Field Study
Source: Vaccines (Basel). 2025 Dec 3;13(12):1221. doi: 10.3390/vaccines13121221 (PMC12737495; doi:10.3390/vaccines13121221)
Supplement: Supplementary file 1 [file vaccines-13-01221-s001.zip › Supplementary Figure S5.pdf]

Epitope #	B-cell conformational Epitopes of P32 protein in	
	The LSDV Neethling LW1959	The SPPV NISKH
1	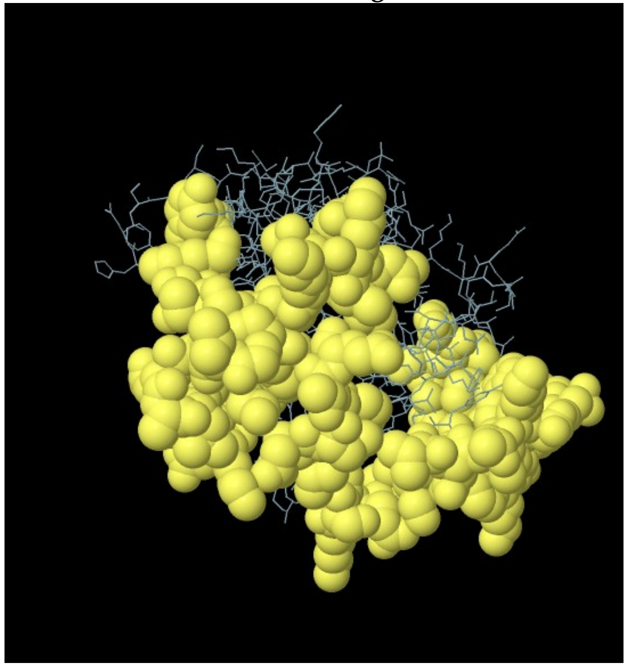	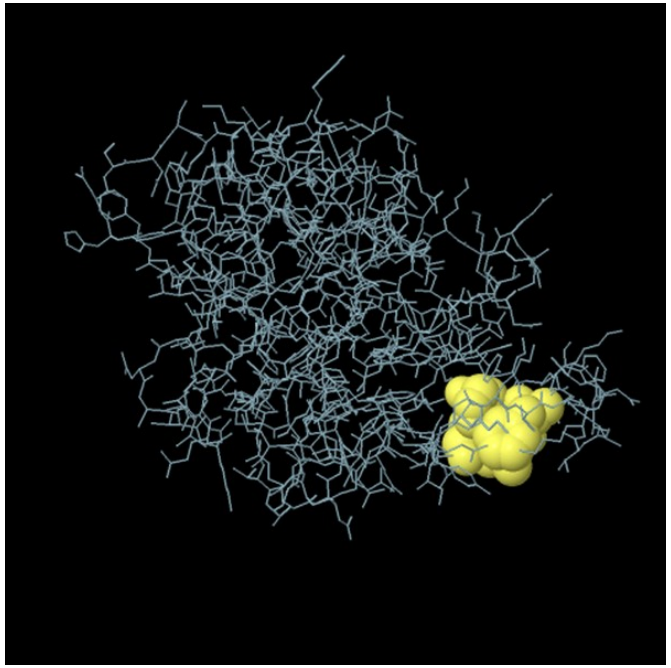

2

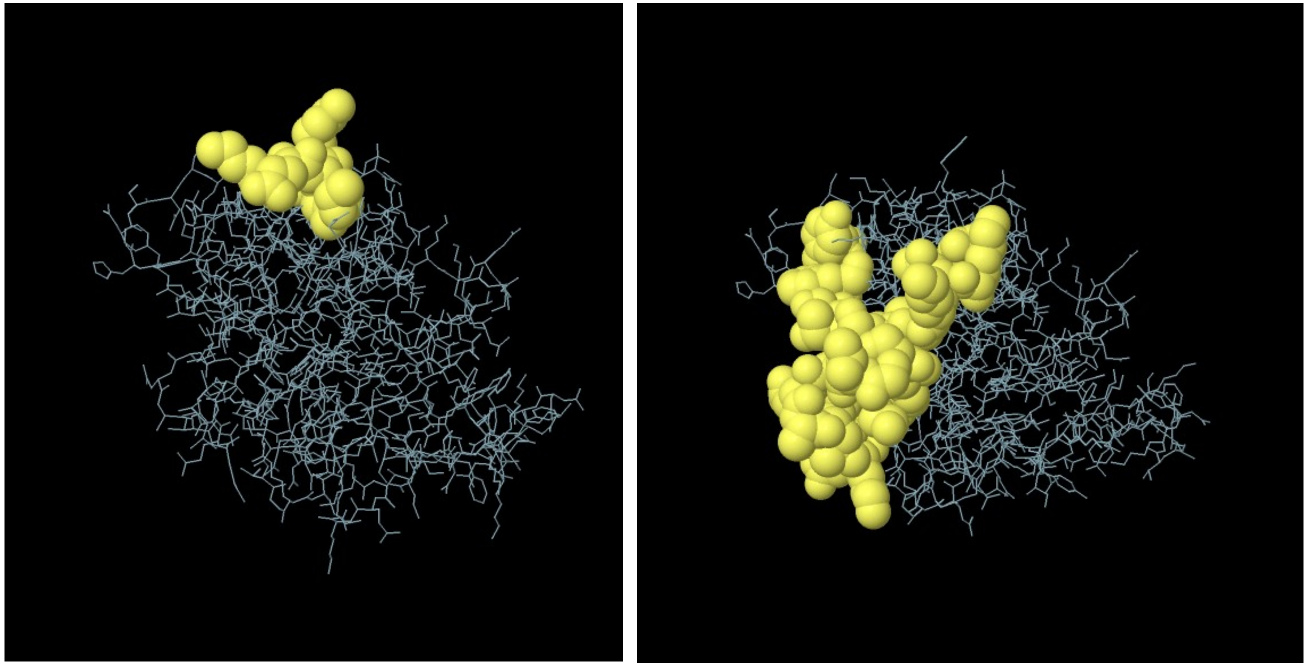

3

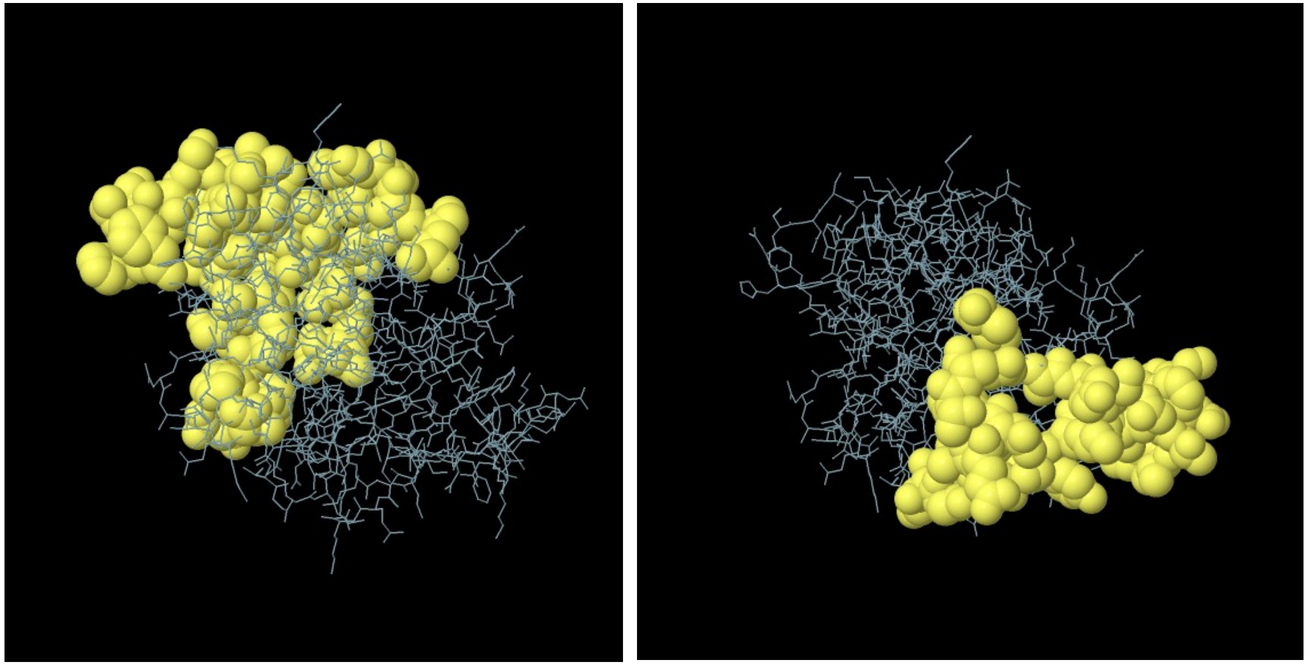

4

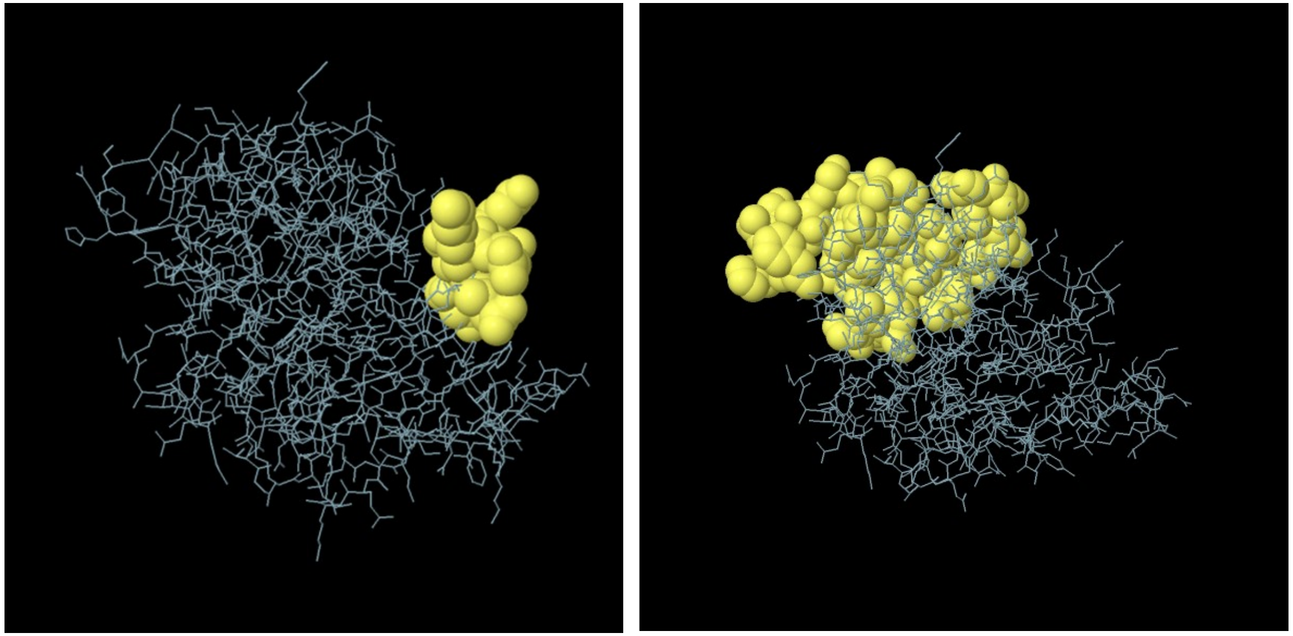

**5**	**Not applicable**	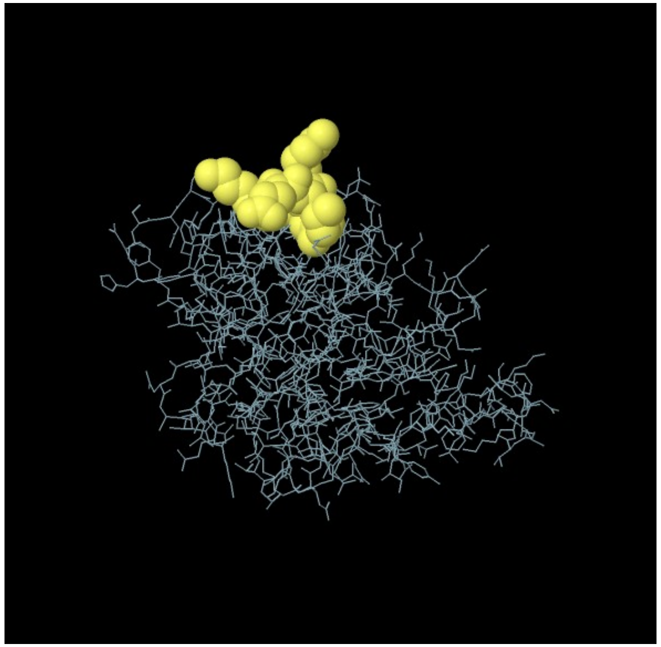

**6**	**Not applicable**	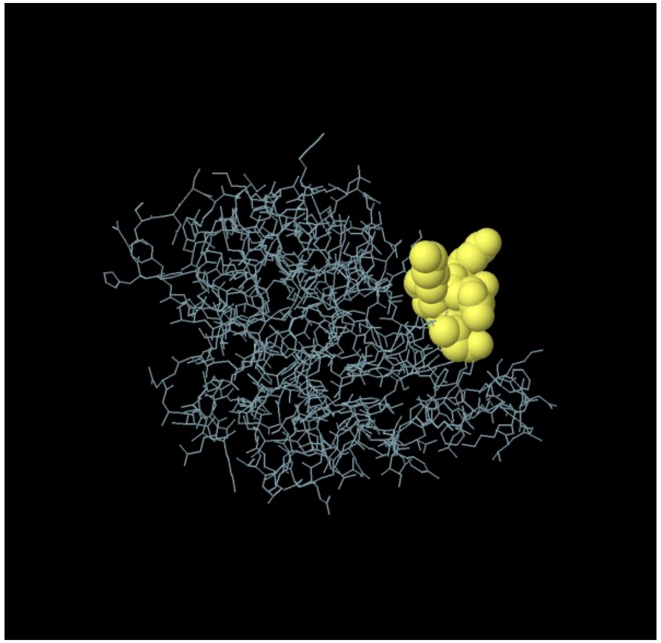

7	Not applicable	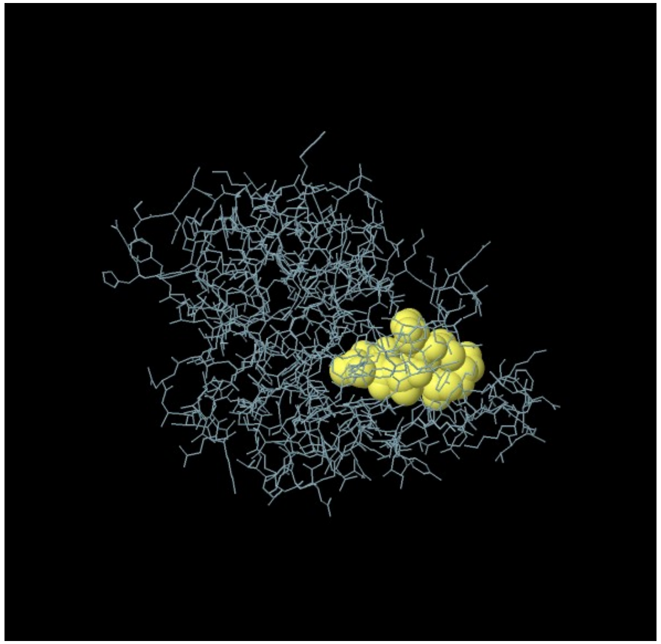

**8**	**Not applicable**	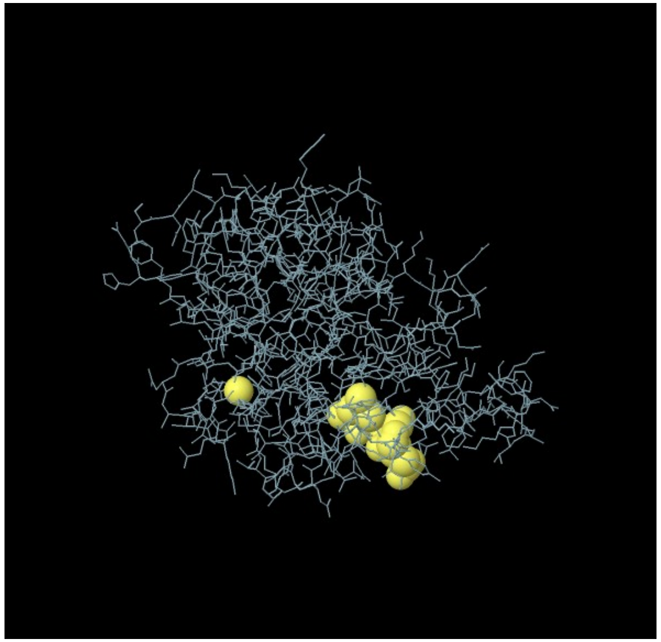

9	Not applicable	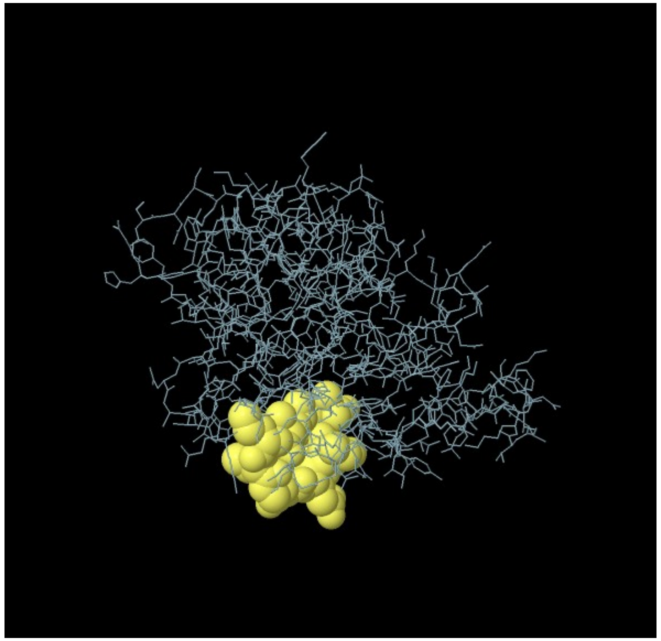

Supplementary Figure S5. The pairwise 3D ribbon diagrams modeling B-cell conformational epitopes residues of P32 protein on the basis of the relevant original amino acid sequences of P32 from either the LSDV Neethling or the SPPV NISCHI strains.
