# Supplementary material for: Humoral Response in Cattle Vaccinated with the Heterologous Sheeppox Virus Vaccine for Protection Against Lumpy Skin Disease: A Field Study
Source: Vaccines (Basel). 2025 Dec 3;13(12):1221. doi: 10.3390/vaccines13121221 (PMC12737495; doi:10.3390/vaccines13121221)
Supplement: Supplementary file 1 [file vaccines-13-01221-s001.zip › Supplementary Figure S6.pdf]

B-cell_1 B-cell_2 B-cell_3

MADIPLYVIPIVGREISDVVPELKSDNDIFYKKVDTVKDFKNSDVNFFFKDKKDISLSYKFLIWEKVEKSGGVENFTEYFSGLCNALCTKEAKSSI

T-cell_2-4 T-cell_5

B-cell_4 B-cell_5 B-cell_6

VKHFSLWKSYADADIKNSENKFIVVIEDDNTLKDLITIHNIIIEMQEKNIDIFQLRETFHNSNSRILFNQENNNFMYSYTGGYDFTLSAYVIRLSS

T-cell_1

B-cell_7 B-cell_8

AIKIINEIIKNKGISTSLSFEMYKLEKELKLNRQVLNDSSKYILHNTKYLSKKRANEMKNGIWNRVGKWMAHRFPDFSYYISHPLVSFFGIFDISI

IGALIILFIIIMIIFDLNSKLLWFLAGMLFTYII

(a)

B-cell_1 B-cell_2 B-cell_3

MADIPLYVIPIVGREISDVVPELKSDNDIFYKKVDTVKDFKNSDVNFFLKDKKDDISLSYKLLIWEKVEKSGGVENFTEYFSGLCNALCTKEA

T-cell_2-4 T-cell_5

B-cell_4 B-cell_5 B-cell_6

KSSIAKHFSLWKSYADADIKNSENKFIVVIEDDNTLKDSIIIHNIIIEMQEKNIDIFQLRETFHNSNSRILFNQENNNFMYSYTGGYDFTLSAYVI

T-cell_1

B-cell_7 B-cell_8

RLSSAIKIINEIIKNKGISTSLSFEMYKLEKELKLNRQVLNDSSKYILHNTKYLSKKRANEMKNGIWNRVGKWMAHRFPDFSYYVSHPLVSFFGIF

DISIIGALIILFIIIMIIFNLNSKLLWFLAGMLFTYII

(b)

Supplementary Figure S6. Positions of the B-cell- and the T-cell epitopes predicted by *in silico* method (http://tools.iedb.org/ellipro/) on the amino acid sequences of either the SPPV vaccine NISKHI (**a**) or the LSDV Neethling vaccine (**b**) strains. The epitopes are numbered sequentially: (i) from 1–8 (B-cell epitopes); and (ii) from 1-5 (T-cell epitopes), throughout the P32 protein. The B-cell epitopes are colored in blue and the T-cell epitopes are highlighted in bold, italic or underlined font/letters and framed in a red box, respectively.
