# Supplementary figures and images for: Humoral Response in Cattle Vaccinated with the Heterologous Sheeppox Virus Vaccine for Protection Against Lumpy Skin Disease: A Field Study
Source: Vaccines (Basel). 2025 Dec 3;13(12):1221. doi: 10.3390/vaccines13121221 (PMC12737495; doi:10.3390/vaccines13121221)

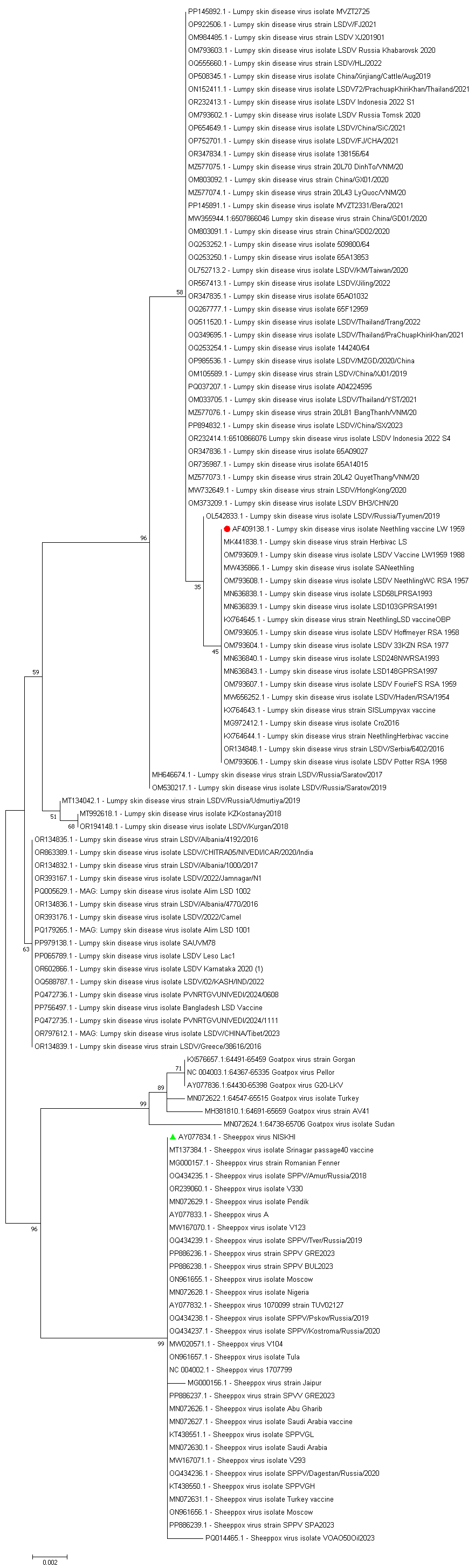

Supplement: Supplementary file 1 [file vaccines-13-01221-s001.zip › Supplementary Figure S3.PNG]
